# A web-based collection of genotype-phenotype associations in hereditary recurrent fevers from the Eurofever registry

**DOI:** 10.1186/s13023-017-0720-3

**Published:** 2017-10-18

**Authors:** Riccardo Papa, Matteo Doglio, Helen J. Lachmann, Seza Ozen, Joost Frenkel, Anna Simon, Bénédicte Neven, Jasmin Kuemmerle-Deschner, Huri Ozgodan, Roberta Caorsi, Silvia Federici, Martina Finetti, Maria Trachana, Jurgen Brunner, Liliana Bezrodnik, Mari Carmen Pinedo Gago, Maria Cristina Maggio, Elena Tsitsami, Wafaa Al Suwairi, Graciela Espada, Anna Shcherbina, Guzide Aksu, Nicolino Ruperto, Alberto Martini, Isabella Ceccherini, Marco Gattorno, for the Paediatric Rheumatology International Trials Organisation (PRINTO) and the Eurofever Project

**Affiliations:** 1 EULAR Centre of Excellence in Rheumatology 2008-2018, IRCCS Istituto Giannina Gaslini, Genoa, Italy; 2 National Amyloidosis Centre, Royal Free Campus, University College Division of Medicine, London, UK; 3 Department of Pediatric Nephrology and Rheumatology, Hacettepe University, Ankara, Turkey; 4 Division of Pediatrics, University Medical Center, Utrecht, The Netherlands; 5 Department of Internal Medicine, Radboudumc Expertise Centre for Immunodeficiency and Autoinflammation, Radboudumc, Nijmegen, The Netherlands; 6 Centre de reference national pour les Arthrites Juveniles, Unite d’Immunologie, Hematologie et Rhumatologie Pediatrique, Universite Paris-Descartes, IMAGINE Institute, Hopital Necker-Enfants Malades, Paris, France; 7 Rheumatologisches Zentrum/Ambulanzfur Autoimmunerkrankungen, Universitatsklinikum Tubingen, Tubingen, Germany; 8 Ic Hastalıkları ABD, Romatoloji BD, Istanbul Universitesi Cerrahpaşa Tıp Fakültesi, Istanbul, Turkey; 9 Department of Pediatrics I, Aristotle University of Thessaloniki, Thessaloniki, Greece; 10 Department fur Kinder-und Jugendheilkunde, Klinikfur Padiatrie I, Padiatrische Rheumatologie, Medizinische Universität Innsbruck, Innsbruck, Austria; 11 Immunology Unit, Hospital de Ninos Ricardo Gutierrez, Buenos Aires, Argentina; 12 Unidad de Reumatología Pediátrica, Hospital Universitario Cruces, Bilbao, Spain; 13 Dipartimento Universitario, Ospedale dei Bambini, Palermo, Italy; 14 Pediatric Rheumatology Unit, 1st Department of Pediatrics, Children’s Hospital Aghia Sophia, University of Athens, Athens, Greece; 15 Department of Pediatrics, King Abdulaziz Medical City, King Saud bin Abdulaziz University for Health Sciences, Riyadh, Kingdom of Saudi Arabia; 16 Seccion Reumatologia, Hospital de Niños Ricardo Gutierrez, Buenos Aires, Argentina; 17 Research Institute for Paediatric Hematology, Moscow, Russia; 18 EgeUniversitesi Tıp Fakultesi, Pediatrik Romatoloji, Izmir, Turkey; 19 Direzione Scientifica, IRCCS Istituto Giannina Gaslini, Genoa, Italy; 20 UOC Medical Genetics, IRCCS Istituto Giannina Gaslini, Genoa, Italy

**Keywords:** Hereditary recurrent fevers, FMF, Caps, Traps, MKD, Infevers, Eurofever, Genotype-phenotype associations

## Abstract

**Background:**

Hereditary recurrent fevers (HRF) are a group of rare monogenic diseases leading to recurrent inflammatory flares. A large number of variants has been described for the four genes associated with the best known HRF, namely *MEFV, NLRP3, MVK, TNFRSF1A*. The Infevers database (http://fmf.igh.cnrs.fr/ISSAID/infevers) is a large international registry collecting variants reported in these genes. However, no genotype-phenotype associations are provided, but only the clinical phenotype of the first patient(s) described for each mutation. The aim of this study is to develop a registry of genotype-phenotype associations observed in patients with HRF, enrolled and validated in the Eurofever registry.

**Results:**

Genotype-phenotype associations observed in all the patients with HRF enrolled in the Eurofever registry were retrospectively analyzed. For autosomal dominant diseases (CAPS and TRAPS), all mutations were individually analyzed. For autosomal recessive diseases (FMF and MKD), homozygous and heterozygous combinations were described. Mean age of onset, disease course (recurrent or chronic), mean duration of fever episodes, clinical manifestations associated with fever episodes, atypical manifestations, complications and response to treatment were also studied.

Data observed in 751 patients (346 FMF, 133 CAPS, 114 MKD, 158 TRAPS) included in the Eurofever registry and validated by experts were summarized in Tables. A total of 149 variants were described: 46 *TNFRSF1A* and 27 *NLRP3* variants*,* as well as various combinations of 48 *MVK* and 28 *MEFV* variants were available.

**Conclusions:**

We provide a potentially useful tool for physicians dealing with HRF, namely a registry of genotype-phenotype associations for patients enrolled in the Eurofever registry. This tool is complementary to the Infevers database and will be available at the Eurofever and Infevers websites.

## Background

Hereditary recurrent fevers (HRF) are a group of autoinflammatory diseases characterized by recurrent fever episodes of variable duration, associated with elevation of acute phase reactants and a number of systemic inflammatory manifestations, mainly involving skin, joints and serosal surfaces [1]. The best known HRF are Familiar Mediterranean Fever (FMF), Cryopyrin-Associated Periodic Syndrome (CAPS), TNF-receptor associated periodic fever syndrome (TRAPS) and Mevalonate-Kinase Deficiency (MKD), caused by mutations in *MEFV*, *NLRP3*, *TNFRSF1A* and *MVK* genes, respectively.

The large number of common variants or polymorphisms in these genes makes assessment of genotype-phenotype association difficult. Furthermore, the possible extent of clinical manifestations associated with fever in HRF is still largely unknown and atypical symptoms may be present. The Infevers database (available at http://fmf.igh.cnrs.fr/ISSAID/infevers/) collects all the reported variants of these genes [2]. Infevers provides a concise description of the clinical picture of first patient(s) reported for each given mutation, but no further genotype-phenotype association. The Eurofever project, supported by the European Agency for Health and Consumers, aims at increasing the knowledge on autoinflammatory diseases [3]. One of the main purposes of the project was to establish an international registry collecting complete demographic, genetic and clinical data of all monogenic autoinflammatory diseases. The aim of the present study is to develop an open web-based registry of genotype-phenotype associations derived from all the patients with HRF enrolled and validated in the Eurofever registry.

## Methods

All patient data were extracted from the Eurofever registry, which has been enrolling patients since November 2009. Independent ethical approval for entering patients in the registry was obtained in the participating countries, in accordance with local requirements. Detailed epidemiological, demographic, molecular and clinical data were collected anonymously. The clinical characteristics included the disease pattern (defined by either recurrent acute episodes, chronic disease or chronic with acute exacerbations), disease manifestations and response to treatment. The Eurofever steering committee has appointed a group of experienced clinicians (SÖ & HÖ for FMF; JF & AS for MKD; HL, MG & PW for TRAPS; BN & JKD for CAPS) for the diagnosis adjudication process. In brief, each adjudication committee had the task to review all anonymized demographic, clinical and genetic information available to confirm/modify/request further information/reject the diagnosis attribution by the enrolling physician.

We created a table for each HRF describing the genotype-phenotype associations observed in all the patients enrolled in the Eurofever registry from November 2009 to November 2014 and validated by the experts. The general features of each cohort of patients have been already described in separate papers [4–7]. For autosomal dominant diseases (CAPS and TRAPS), all mutations were reported individually. For autosomal recessive diseases (FMF and MKD), homozygous and all combinations of heterozygous variants were described. As complex alleles could not be excluded, combinations presented do not imply in any case compound heterozygous conditions.

A separate table was devoted to the description of the clinical phenotype of patients with an incomplete genotype, such as heterozygous patients with autosomal recessive diseases or patients carrying low-penetrance mutations or variants/polymorphisms of uncertain pathogenic significance. In particular, according to the recent literature, we considered of unknown significance the p.E148Q and p.P369S variants of the *MEFV* gene; the p.P46L, p.R92Q and intronic variants, exept c.193-14G > A, of the *TNFRSF1A* gene; the p.V198 M and p.Q703K of the *NLRP3* gene. The p.R202Q of the *MEFV* gene was also included in Table 6, despite this variant is considered a common and neutral polymorphism that should not be even reported [8–14].

Patients with heterozygous mutations of the *MVK* gene were included in the study only after the demonstration of a reduced mevalonate kinase enzyme activity in leukocytes or fibroblasts, or elevated urinary mevalonic acid excretion [15]. For each variant or genotype, the following items were shown: number of patients, mean age of onset, disease course (recurrent or chronic), mean duration and frequency of fever episodes, prevalent clinical manifestations associated with fever episodes, less common manifestations, complications and response to treatments. Less common manifestations were defined as symptoms present in less than 30% of patients carrying a given genotype.

Treatment response was defined as either complete (absence of clinical manifestations with normalization of inflammatory markers), partial (general amelioration of the clinical picture according to the judgement of the enrolling physician without a complete normalization of the clinical manifestations and/or systemic inflammation), or failure (lack of response according the judgment of the enrolling physician). The distinction between on demand and continuous treatment was also possible.

## Results

A total of 751 patients (346 FMF, 114 MKD, 158 TRAPS, 133 CAPS) were enrolled in the analysis (Table 1). In total, 149 variants of the four genes associated with HRF are reported.

**Table 1:** Demographic characteristics of the patients

Disease	Fmf	Mkd	Traps	Caps
N° of patients	346	114	158	133
N° of countries	28	12	18	16
N° of variants	28	48	46	27
N° of variants of unknown significance	2	–	5	2
N° of combinations	33	50	–	–
- Heterozygous	26	46	–	–
- Homozygous	7	4	–	–
Age at onset (years: median, range)	3 (0–67)	0.5 (0–11)	4.3 (0–63)	0.8 (0–45)

A summary of the main clinical features associated with homozygous or more than one heterozygous variants combinations of 19 *MEFV* and 47 *MVK* mutations are described in Tables 2 and 3. Tables 4 and 5 show the main clinical features of patients carrying 44 *TNFRSF1A* and 25 *NLRP3* mutations, respectively. In Table 6, data of 210 patients with an incomplete or not confirmatory genotype are also reported. In case of autosomal recessive diseases, we reported as first mutation the variant nearest to the proximal end of the coding sequence.

**Table 2:** Genotype-phenotype associations in patients with FMF

Gene region	First mutation	Second mutation	N° pt	Age at onset (years; average, range)	Disease course (n. of patients)	Prevalent clinical manifestations (n. of patients)	Less common manifestations (n. of patients)	Treatment response (n. of patients)	Complications (n. of patients)
Exon 2	S108R	V726A	1	0	Recurrent	Fever or low fever episodes of 4 days, 12 episodes/year, with exudative and erythematous pharyngitis, arthralgia, myalgia, enlarged bilateral and painful cervical lymphonodes, periorbital pain and conjunctivitis, vomiting, oligoarthritis, chest pain, headache in the morning or anytime, constipation and abdominal pain.		Partial response to steroids. Complete response to colchicine.	
	E148Q	M680 L	1	6	Recurrent	Fever or low fever episodes, 3/year, with erythematous pharyngitis, arthralgia, abdominal pain and headache.		Complete response to colchicine.	
		M694 V	15	5 (0–27)	Recurrent	Fever episodes of 2–3 days, 13/year, with regular pattern, chills at onset (6), associated with abdominal pain (12), arthralgia (10) and chest pain (5).	Vomiting, headache (4), diarrhea, erythematous pharyngitis, aphtous stomatitis (3), monoarthritis, erysipelas-like erythema, costipation, myalgia (2), general or cervical bilateral enlarged lymphonodes, oligoarthritis, maculopapular rash, pseudofollicolitis, oral herpetic-like lesions, myositis, fascitis, tenosynovitis,bone pain, aseptis peritonitis, pleurisy, urethritis/cystitis and hydrocephalus (1). Episodes induced by stress (2), menstruation, fatigue, cold and vaccins (1)	Patial response to NSAIDs (3). Partial response (5) or complete (9) to colchicine. One patient responded partially to adalimumab. One patient presented complete response to steroids but developed a fulminant hepatitis A and vertebral collapse as side effect. After liver transplantation, he started cyclosporine and micophenolatemofetil with complete response.	
		V726A	5	3 (2–7)	Recurrent	Fever (4) or low fever (2) episodes of 2 days, 21/year, with abdominal pain (4) and arthralgia (2).	Chest pain, hepatomegaly, splenomegaly and maculo-papular rash (1).	Partial response to NSAIDs, steroids, colchicine (2) and cimetidine (1). Complete response(1) or failure (1) with colchicine.	
		A744S	4	35 (14–51)	Recurrent	Low fever (2) or fever (1) of 3–4 days, 9/year, with abdominal pain, arthralgia (3), erysipelas-like erythema, myalgia (2), monoarthritis, conjunctivitis and chest pain (1).		Partial response to NSAIDs (2) or steroids (1)on-demand. Complete response to colchicine (2).	
		R761H	4	5 (1–16)	Recurrent	Low fever of 3 days, 16/year, induced by exercise (2), with arthralgia (4), chills, myalgia, vomiting, abdominal and chest pain (3), pleurisy and diarrhea (2).	Enlarged cervical lymphonodes, headache in the morning or anytime, polyarthritis and dysmenorrhea (1).	Complete response to colchicine (4).	
	E167D	M694 V	1	1	Recurrent	Fever episodes of 2 days, 18/year, induced by infections, with conjunctivitis and abdominal pain.		Complete response to colchicine.	
	E225G	M694 V	1	1	Recurrent	Fever episodes of 5 days, 12/year, with arthralgia, myalgia, pseudofolliculitits, erythematous pharyngitis, abdominal and chest pain.		Partial response to steroids on-demand. Complete response to colchicineasmantainance treatment.	
Exon 5	F479 L	V726A	1	4	Recurrent	Fever episodes of 3 days, 15/year, with abdominal pain and conjunctivitis.		Complete response to colchicine.	
Exon 9	I591T	V726A	1	5	Recurrent	Fever episodes of 2 days, 5/year, induced by stress, fatigue and exercise, with diarrhea, constipation and abdominal pain.		–	
Exon 10	M680IGA	M680IGA	3	4 (0–8)	Recurrent	Fever or low fever episodes of 1–2 days, 5/year, with erythematous pharyngitis, myalgia, arthralgia (2) and abdominal pain (3).	Diarrhea, enlarged cervical bilateral lymphonodes (1).	Complete response to NSAIDs on-demand (1). Complete response to colchicine as maintenance therapy (3).	
		M694 V	2	2	Recurrent	Fever episodes of 2 days, 6/year, induced by cold and exercise, with regular pattern (1), associated with maculo-papular rash, myalgia, abdominal pain, chest pain, costipation (2), exudative or erythematous pharyngitis, urticarial rash, arthralgia, gastrointestinal bleeding, hepatosplenomegaly, aseptic peritonitis, bone pain, pericarditis, periorbital oedema and conjunctivitis(1).		Adenotonsillectomy failed. Complete responsetocolchicine as maintenance therapy(1).	
		V726A	4	5 (1–7)	Recurrent (3). Continuous and recurrent (1).	Fever or low fever episodes of 4 days, 12/year, sometimes induced by cold and stress (2), with abdominal pain (4), vomiting and arthralgia (2).	Myalgia, mono/oligoarthritis, diarrhea or constipation, enlarged cervical lymphonodes, hepatomegaly, chest pain, aseptic peritonitis and costipation, headache, vertigo (1).	Complete (1) or partial (2) response to colchicine. Tonsillectomy, steroids and NSAIDs failed (1). Partial response to NSAIDs (1).	Peritoneal adhesions (1).
		R761H	1	0	Recurrent	Fever or low fever of 1 days, 12/year, sometimes induced by vaccination, with regular pattern, chills, urticarial rash and abdominal pain.		Complete response to NSAIDs or steroids on-demand. Complete response to colchicine as maintenance therapy.	
	M680IGC	M680IGC	5	3 (1–6)	Recurrent	Fever episodes of 2 days, 18/year, with regular pattern, associated with exudative pharyngitis, enlarged cervical bilateral and painful lymphonodes, erysipelas-like erythema, splenomegaly, headache (2), myalgia, arthralgia, vomiting, diarrhea, pericarditis (3), aseptis peritonitis and pleurity (4), abdominal and chest pain (5).	Oligoarthritis and tenosynovitis, costipation, gastrointestinal ulcers, hepatomegaly, painful orchitis (1).	Complete response to colchicine as maintenance therapy (5).	Delayed puberty, peritoneal adhesions, and severe infections (1).
		T681I	1	11	Recurrent	Fever episodes of 3 days, 3/year, with erythematous pharyngitis, chest pain, pleurisy, arthralgia, myalgia and abdominal pain.		Complete response to colchicine as maintenance therapy.	
		M694 V	31	2 (0–30)	Recurrent	Fever or low fever episodes of 3 days, 15/year, with regular pattern, with arthralgia, abdominal pain (21), myalgia (17), oligoarthritis (16), chest pain (15), pleurisy (12), vomiting (10) and aseptic peritonitis (9).	Headache (7), erysipelas-like erythema, diarrhea, chills at fever onset (6), exudative (1) or erythematous pharyngitis, tenosynovits, enlarged bilateral and painful cervical lymphonodes (5), hepatomegaly, pericarditis (4), urticarial rash, bone pain, poliarthritis, gastrointestinal ulcers (3), pneumonia, persistent cough, monoarthritis (2), hemoptysis, purpuric or maculopapular rash, myositis, periorbital oedema, uveitis anterior, constipation, urethritis/cistictis, enuresis, hypoplasia of left kidney (1). Episodes induced by cold (8), stress (3), exercise, food (2), hunger, trauma, travel, infection (1).	Complete (15) or partial (11) response to colchicine. Partial (6) or complete (2) response to NSAIDs. Partial response to steroids, methotrexate (3),sulphasalazine (1). One patient responded completely to allopurinol.	Flexion contractures, osteoporosis (5), bone alteration, spondilloarthropathy, renalamyloidosis (2), bone deformity, atrophy of the muscles of the lower extremities, cataract, cranial neuropathy, macrophage activation syndrome (1).
		V726A	4	5 (1–12)	Recurrent	Fever episodes of 3 days, 13/year, with myalgia (2), chest pain (3) and abdominal pain (4).	Arthralgia, oligoarthritis, vomiting, constipation, gastrointestinal ulcers, aseptic peritonitis, pleuritis, pneumonia, headache, urethritis/cistitis (1).	Complete (3) response to colchicine, that failed in one patient. Partial response to NSAIDs (1). Complete response to steroids (1) or anakinra (1).	
		R761H	2	17 (4–30)	Recurrent	Fever episodes of 2–3 days, 9/year, with chest pain (2), pleurisy, arthralgia, myalgia, abdominal pain (1).		Complete response to colchicine as maintenance therapy.	
	M680 L	M680 L	1	1,5	Recurrent	Fever episodes of 6 days, 10/year, with regular pattern and chills at fever onset, associated with arthralgia, oligoarthritis, vomiting and diarrhea, abdominal pain.		Partial response to NSAIDs; complete response to tocolchicine as maintenance therapy. Bilateral sacro-iliac joints steroid injection and tonsillectomy.	
	I692del	M694 V	1	2	Recurrent	Fever or low fever episodes of 2 days, 18/year, with abdominal pain.		Complete response to colchicine as maintenance therapy.	
	M694I	M694I	4	3 (1–5)	Recurrent	Fever episodes of 8 days, 9/year, with vomiting and costipations, arthralgia, chest pain (2) and abdominal pain (4).	Erythematous pharyngitis with enlarged cervical bilateral lymphonodes, chills at fever onset, myalgia, monoarthritis or oligoarthritis, conjuntivitis, diarrhea, aseptic peritonitis, pneumonia, headache (1).	Partial response to NSAIDs (1) or colchicine (2). One patient with complete response to colchicine (1).	Peritoneal adhesions, occlusion/sub-occlusion (1).
		M694 V	6	3 (1–30)	Recurrent	Fever episodes of 2–3 days, 18/year, with abdominal pain, arthralgia (5), chest pain (4), myalgia, headache (3) and vomiting, diarrhea, pleurisy (2).	Urticarial rash, erysipelas-like erythema, psoriasis, eczema, monoarthritis, tenosynovitis, GI ulcers, generalized or cervical bilateral enlargement lymphonodes, hepatomegaly and splenomegaly, pericarditis, pneumonia (1). Episodes induced by food, fatigue and vaccin (1).	Only two patients respond completely to colchicineas maintenance therapy. Response to NSAIDs partial (1) or failed (1). Steroids failed (1). One patient responds completely to anakinra, with urticarial rash at start, disappeared.	Peritoneal adhesions (1).
	M694 V	M694 V	96	3 (0–17)	Recurrent	Fever episodes of 2 days, 14/year, with abdominal pain (86), arthralgia (79), myalgia (57), chest pain (52), oligoarthritis (34), vomiting (32), chills at fever onset and diarrhea (30).	Pleurisy (27), pericarditis (20), exudative (7) or erythematous (18) pharyngitis, monoarthritis (25), erysipalas-like erythema, aseptic peritonitis (20), splenomegaly (16), hepatomegaly (15), seasonal changes of frequency of fever episodes (13), enlarged bilateral and painful cervical lymphonodes (12), maculo-papular rash and costipation (10), tenosynovitis (9), headache anytime (25) or only in the morning (7), flexion contractures and gastrointestinal ulcers (6), poly/oligoarthritis, conjunctivitis (5), gonadal pain (4), anal/perianal ulcers, aphtous stomatitis and urticarial rash, urethritis/cistitis (3), papulopustular lesions and palpable purpura, bone pain, GI bleeding, pneumonia and persistent cough, vertigo (2), hemoptysis, generalized enlargement, psoriasis and ichthyosiform rash, herpetic-like oral lesions, hemorrhagic rash, erythema nodosum, mesadenitis, optic neuritis, seizures and cranial nerve palsy (1). Episodes inducted by cold (18), stress (8), infect (4), fatigue (3), travael and exercise (2), food and vaccin (1).	Response to NSAIDs partial (27), complete (8) or failed (4). Response to steroids partial (7), complete (4) or failed (6). Response to colchicine partial (38), complete (38) or failed (3). Response to sulphasalazine complete (1) and partial (2). Response to methotrexate complete (1), partial (4) and failed (1). One patient responds partially to cyclosporine. One patient responds partially and one completely to anakinra but develops skin reaction, pustular lesion on the injection site. One patient responds partially to canakinumab. One patient responds partially to etanercept but develops a worsening of kidney.	Peritoneal adhesions (8), occlusion, osteoporosis non steroids-related, bone deformity (3), gut perforation and bone alteration (2), bone erosions, hyperostosis and cholelithiasis (1).
		M694del	1	0	Recurrent	Fever episodes of 1 days, 12 per year, with abdominal pain and arthralgia.		Partial response to Colchicine.	
		M694 L	1	6	Recurrent	Fever or low fever episodes of 7 days, 12/year, with bone and abdominal pain, constipation.		Complete response to maintenance with colchicine.	
		K695R	2	7	Recurrent	Fever episodes, 7/year, with arthralgia, constipation and abdominal pain (2).		Partial response to sulphasalazine (1), complete response to colchicine (2).	
		V726A	29	3 (0–45)	Recurrent	Fever or low fever episodes of 2–3 days, 12/year, with abdominal pain (25), arthralgia (20), myalgia, chest pain (16), vomiting (14).	Diarrhea (8), chills at fever onset (7), bilateral and painful cervical enlargement lymphonodes, oligoarthritis(6), pericarditis, pleurisy, exudative pharyngitis (5), aseptic peritonitis, headache (4), constipation, GI ulcers, erythematous pharyngitis, urethritis/cistitis (3), erysipelas-like erythema, hepatomegaly, generalized enlargement lymphonodes, bone pain, monoarthritis (2), splenomegaly, maculo-papular rash, aphtous stomatitis, vaginal hydrocele, periorbital pain and conjunctivitis or uveitis anterior and choreoretinitis, aseptis meningitis, gonadal enlarged, height related ipertension (1). Episodes induced by cold (2), infection or fatigue (1).	Complete (3) or partial (5) response to NSAIDs. Complete (1) or partial (2) response to Steroids. Complete (14) or partial (10) response to Colchicine: two patients developed diarrhea. Partial response to tonsillectomy (4).	
		R761H	6	5 (1–11)	Recurrent	Fever or low fever episodes of 2 days, 10/year, with abdominal pain (6), vomiting (4), arthralgia, myalgia, constipation, chest pain (3), pericarditis and pleurisy (2).	Erythematous pharyngitis, urticarial rash, aseptic peritonitis, bilateral cervical enlargement lymphonodes, hepatomegaly, persistent cough, hemoptysis, headache and orchitis (1). Episodes induced by cold, stress, food (1).	Complete response to colchicine (4), steroids (1) and NSAIDs (1).	
	V726A	V726A	2	0	Recurrent	Fever episodes of 4–5 days, 11/year, with seasonal changes of frequency, stress or fatigue as triggers, with abdominal pain, arthralgia, myalgia, enlarged bilateral cervical lymphonodes, hepatomegaly, splenomegaly and seizures (1).		One patient responds partially to colchicine and develops alopecia areata. Tonsillectomy and adenoidectomy failed (1).	
	A744S	A744S	1	3	Recurrent	Fever or low fever episodes of 11–15 days, 3/year, with abdominal pain, chest pain, pericarditis, vomiting, diarrhea, erysipelas-like erythema, arthralgia, myalgia, fatigue and malaise.	Partial response to NSAIDs or steroids or colchicine. Complete response to etanercept as maintenance therapy.

**Table 3:** Genotype-phenotype associations in patients with MKD

Gene region	First mutation	Second mutation	N° pt	Age of onset (years; average, range)	Disease course (*n*. of patients)	Prevalent clinical manifestations (n. of patients)	Less common manifestations (*n*. of patients)	Treatment response (*n*. of patients)	Complications (n. of patients)
Exon 2	c.1_26del	V377I	1	3	Continous and recurrent	Fever episodes of 7 days, 10/year, with aphthous stomatitis, exudative or erythematous pharyngitis, abdominal pain, arthralgia and myalgia.		Partial response to NSAIDs and complete response to steroids on demand.	
	H20N	R215Q	2	At birth	Continuous	Persistent high-grade fever with polyarthritis, arthralgia, persistent cough and occasional low-grade fever, erysipelas-like erythema, papulopustular lesions, tenosynovitis, lymphadenopathy, chest pain, pericarditis, pneumonia, myalgia, bone pain, fatigue and malaise.		Partial response to NSAIDS on-demand. Partial response to steroids, cyclosporine, biphosphonates and anakinra as maintenance therapy. Colchicine, infliximab, methotrexate, sulphasalazine, azathioprine, statins, tacrolimus and etanercept failed.	Bone alterations and erosions, flexion contractures, osteolytic lesions, osteoporosis, cataract, impaired vision.
				At birth	Recurrent	Fever episodes of 5 days, 12/year, with acne, diarrhea, hepatomegaly, splenomegaly, lymphadenopathy, polyarthritis and occasional abdominal pain, vomiting, aseptic peritonitis, exudative pharyngitis, scleritis, pneumonia, persistent cough, arthralgia, myalgia, fatigue, malaise and bone pain.		Partial response to NSAIDs on-demand. Complete response to anakinra and partial to steroids, biphosphonates, intravenous immunoglobulin and etanercept as maintenance therapy. Colchicine, sulphasalazine, methotrexate, azathioprine and statins and tonsillectomy failed.	Bone alterations and erosions, flexion contractures, osteolytic lesions, osteoporosis, cataract, peritoneal adhesions, sub−/occlusions, gut perforations and recurrent inguinal hernia.
	H20P	V377I	3	4	Recurrent	Fever episodes of 4 days, 8/year, with myalgia, vomiting,maculo-papular rash, abdominal pain, fatigue, malaise, diarrhea (3), exudative or erythematous pharyngitis, arthralgia, lymphadenopathy, mood disorders, headache (2), hepatosplenomegaly (1). Occasional episodes of aphthous stomatitis with or without fever.		Partial response to NSAIDs (1) or anakinra (1) on-demand. Steroids (1) failed. Colchicine (2) or statins (1) failed as maintenance therapy. No response to tonsillectomy (1).	Neuropathy (1).
	V20R	V377I	1	1	Recurrent	Fever episodes, 10/year, with urticarial rash, lymphadenopathy, arthralgia and occasional vomiting and diarrhea.		Partial response to steroids on-demand.	
Exon 3	H44Qfs*35	V377I	1	7	Recurrent	Fever episodes, 6/year, with maculo-papular rash, arthralgia, myalgia, abdominal pain, vomiting, diarrhea, fatigue, malaise and weith loss.		On-demand Steroids failed. Partial response to etanercept and azithromicine as maintenance therapy. Colchicine and anakinra failed. Complete response to Infliximab.	
	W62*	V377I	1	At birth	Recurrent	Fever episodes of 6 days, 24/year, with abdominal pain, fatigue and malaise, occasional with urticarial rash, arthralgia, myalgia, vomiting, diarrhea, gastrointestinal bleeding. Persistence of hepato-splenomegaly, mood disorders, generalized enlarged lymphadenopathy.		On-demand NSAIDs failed. Partial response to steroids on demand.	
Exon 4	Y114Ifs*71	N205D	1	0.1	Recurrent	Fever episodes of 13 days, 17/year, with occasional abdominal pain and splenomegaly. Occasional episodes of erythema marginatum, arthralgia, myalgia and oligoarthritis with or without fever.		Partial response to steroids and anakinra as maintenance therapy. Colchicine and etanercept failed.	
	Y116H	V377I	1	0.2	Recurrent	Fever episodes of 5 days, 22/year, with aphthous stomatitis, arthralgia, headache, abdominal pain, diarrhea, lymphadenopathy and occasional urticarial rash.		Partial response to colchicine and anakinra as maintenance therapy. Partial response to tonsillectomy.	
	I119M	A148T	1	2	Recurrent	Fever episodes of 7 days, 24/year, with always arthralgia, abdominal pain, vomiting, diarrhea and lymphadenopathy. Presence of headache with or without fever.		Partial response to NSAIDs or steroids on-demand. Partial response to etanercept as maintenance therapy.	
	R124W	V377I	1	2	Recurrent	Fever episodes of 4 days, 24/ years, with arthromialgia, myalgia, malaise and occasional abdominal pain, diarrhea, vomiting, erythematous pharyngitis, cervical and painful lymphadenopathy.		Complete response to steroids on demand. No response to tonsillectomy.	
Exon 5	V132I	V377I	1	0.4	Recurrent	Fever episodes of 8 days, 13/year, with exudative and erythematous pharyngitis, arthralgia, myalgia, abdominal pain, lymphadenopathy and occasional splenomegaly.		Partial response to NSAIDs or steroids. Partial response to colchicine and etanercept as maintenance therapy.	
	A141Rfs*18	A334T	1	At birth	Recurrent	Fever episodes of 4 days, 12/year, with aphthous stomatitis, arthralgia, myalgia, abdominal pain, vomiting, diarrhea, lymphadenopathy, fatigue, malaise and occasional erythematous pharyngitis, maculo-papular rash and mood disorders. Episodes of oligoarthritis and headache with or without fever.		Partial response to NSAIDs. Anakinra failed as maintenance therapy.	Impaired vision, cerebellar syndrome, retinitis pigmentosa, mental retardation.
	A147T	V377I	1	0.05	Recurrent	Fever episodes of 3 day, with occasional vomiting, diarrhea, aphthous stomatitis, maculo-papular rash and lymphadenopathy.		Partial response to etanercept. No response to tonsillectomy.	
	C152Wfs*6	V377I	1	0.2	Recurrent	Fever episodes of 2 days, 24/year, with vomiting, diarrhea, painful and enlarged cervical lymphadenopathy, with occasional gastrointestinal bleeding. Abdominal pain and constipation with or without fever.		Partial response to NSAIDs on-demand. On-demand Steroids and etanercept as maintenance therapy failed.	
	P165L	I268T	2	At birth	Continuous	Persistent maculo-papular rash, vomiting, diarrhea, lymphadenopathy, hepatosplenomegaly, malaise and fatigue with occasional episodes of aphthous stomatitis, exudative pharyngitis, arthralgia, myalgia, oligo-arthritis, abdominal pain, aseptic peritonitis and pneumonia with persistent cough, headache and mood disorders.	Occasional episodes of osteitis.	Partial response to steroids on-demand and anakinra as maintenance therapy. Etanercept, statins and montelukast failed. No response to adenotonsillectomy.	Peritoneal adhesions, occlusion/sub-occlusion, venous thrombosis, severe infections.
				0.3	Recurrent	Fever episodes of variable length, 12/year, with occasional exudative and erythematous pharyngitis, maculo-papular rash, palpable purpura, arthralgia, myalgia, mono-arthritis, vomiting, abdominal pain, diarrhea, constipation, lymphadenopathy, headache, fatigue and malaise. Presence of acne with or without fever.		On-demand NSAIDs failed. Partial response to steroids and anakinra on-demand. Statins failed as maintenance therapy.	Mental retardation.
		V377I	2	0.7	Recurrent	Fever episodes of 4 days, 9/year, with abdominal pain, lymphadenopathy, fatigue, malaise (2), diarrhea, arthralgia, headache, aphthous stomatitis, maculo-papular rash (1).		Partial response to NSAIDs on-demand (2). Partial response to colchicine (1) as maintenance therapy. No response to tonsillectomy.	
	N166I	G336S	1	5	Recurrent	Fever episodes of 7 days, 12/year, with occasional aphthous stomatitis, exudative and erythematous pharyngitis, arthralgia, myalgia, abdominal pain, diarrhea, aseptic peritonitis, lymphadenopathy, fatigue and malaise. Presence of occasional episodes of pseudo-folliculitis, papulo-pustular lesions, peri-anal lesions and hepatomegaly with or without fever.		Partial response to colchicine as maintenance therapy.	
Exon 6	W188*	V337I	1	0.5	Recurrent	Fever episodes of 6 days, 24/years, with vomiting, abdominal pain, diarrhea, cervical painful lymphadenopathy and occasional maculo-papular rash, erythematous pharyngitis, arthralgia, myalgia. Persistence of fatigue and malaise. Since about 12 years of age just gastrointestinal symptoms during attacks.		Partial response to NSAIDs or steroids on demand. Partial response to tonsillectomy.	
	Q190fs	V377I	1	11	Recurrent	Fever episodes, 13/year, with lymphadenopathy, vomiting, abdominal pain, erythematous pharyngitis and occasional aphthous stomatitis and arthralgia.		Partial response to NSAIDs on-demand. Partial response to colchicine as maintenance therapy.	
	V203 fs	V377I	2	0.3	Recurrent	Fever episodes of 3 days, 16/year, with urticarial rash, arthralgia, myalgia, abdominal pain, malaise and occasional lymphadenopathy (2), diarrhea, aphthous stomatitis, exudative and erythematous pharyngitis, maculo-papular or migratory rash, fatigue, mood disorders, palpable purpura, pleurisy, pneumonia, hepatomegaly, splenomegaly (1). Moreover presence of occasional conjunctivitis, vomiting, diarrhea, persistent cough and headache with or without fever (1).		Partial response (1) or failure (1) to NSAIDs and partial to steroids (2) or colchicine (1) on-demand. Cyclosporine and statins (1) failed as maintenance therapy.	Cataract, cerebral nerve palsy and mental retardation (1).
	V203A	I268T	1	At birth	Continous and recurrent	Fever episodes of 7 days, 12/year, with aphthous stomatitis, erythematous rash, erysipelas, arthralgia, diarrhea, generalized and painful lymphadenopathy, hepato-splenomegaly, fatigue, malaise and headache.		Partial response to NSAIDs or steroids on demand. Partial response to steroid as maintenance therapy.	Peritoneal adhesions.
	N205D	V312A	1	At birth	Recurrent	Fever episodes of 5 days, 6/year, triggered by stress, with erythematous pharyngitis, arthralgia, myalgia, abdominal pain and sometimes aphtous stomatitis, ulcers at genitalia, vomiting, diarrhea, generalized lymphadenopathy, fatigue and malaise. Headache and conjunctivitis with our without fever.		Partial response to NSAIDs on-demand. Steroids failed.	
Exon 7	G211E	V337I	1	0.7	Recurrent	Fever episodes of 6 days, 14/year, with palpable purpura. Occasional maculo-papular or urticarial or vasculitic rash, abdominal pain, vomiting, diarrhea, enlarged and painful lymphadenopathy, aphthous stomatitis, arthralgia, myalgia, fatigue and malaise with or without fever.	Periorbital edema.	Complete response to NSAIDs or steroids on-demand. Azathioprine failed.	
	R215Q	V377I	3	At birth	Recurrent	Fever episodes of 6 days, 12/year, with abdominal pain, diarrhea, enlarged lymphadenopathy (3), headache, erythematous pharyngitis, painful lymphadenopathy, vomiting, arthralgia (2), maculo-papular rash, splenomegaly, pneumonia, urticarial rash, myalgia, fatigue, malaise (1).	Aphthous stomatitis and gastrointestinal ulcers during fever episodes (1).	Partial response to NSAIDs (2) on-demand. Complete (1) or partial (1) response to steroids. Partial response to rilonacept (1) as maintenance therapy. No response to tonsillectomy (1).	
	c.632-1G > C	V377I	1	2	Recurrent	Fever episodes of 5 days, 14/year, with aphthous stomatitis, pseudofolliculitis, ulcers at genitalia, arthralgia, myalgia, anterior uveitis, episcrleritis, vomiting, diarrhea, abdominal pain, painful cervical lymphadenopathy, splenomegaly, fatigue, malaise and mood disorders.		On-demand NSAIDs or steroids failed. Partial response to anakinra and etanercept as maintenance therapy.	Macrophage activation syndrome.
Exon 8	P228P	V377I	1	2	Recurrent	Fever episodes of 6 days, 7/year, sometimes with enlarged cervical lymphadenopathy, abdominal pain, diarrhea, urticarial rash, fatigue and headache,		Partial response to etanercept as maintenance therapy.	Renal failure.
	T237S	T237S	1	–	Recurrent	Fever episodes of 5 days, 20/year. Episodes of vomiting, abdominal pain, diarrhea, arthralgia and cervical lymphadenopathy with or without fever.		Colchicine failed as maintenance therapy. Partial response to anakinra.	
		I268T	1	At birth	Continous	Fever, malaise, weight loss, maculo-papular rash, vomiting, abdominal pain, diarrhea, gastrointestinal bleeding, aseptic peritonitis, hepato-splenomegaly and episodes of pneumonia.	Ascites, cholestasis.	Partial response to steroids as maintenance therapy.	Peritoneal adhesion, sub/occlusion. Death due to acute respiratory distress syndrome.
	L246P	V337I	1	At birth	Recurrent	Fever episodes of 3 days, 8/year, with exudative and erythematous pharyngitis, generalized and painful enlarged lymphadenopathy, abdominal pain, diarrhea and, sometimes, vomiting, bone pain, arthromyalgia, fatigue, malase, weight loss. Acne and aphthous stomatitis with or without fever.		Partial response to NSAIDs or steroids on-demand.	
Exon 9	L264F	V377I	3	0.6	Recurrent	Fever episodes of 5 days, 13/year, with occasional abdominal pain, maculo-papular rash, aphthous stomatitis, diarrhea, exudative (3) and erythematous pharyngitis, lymphadenopathy, malaise, splenomegaly, fatigue, arthralgia (2), headache hepatomegaly, myalgia (1).	Recurrent pericarditis (1).	Partial response to steroids on-demand (3). Partial response to anakinra as maintenance therapy (1). Partial response (2) or failure (1) to NSAIDS on-demand.	
	L264Sfs*2	V377I	1	At birth	Recurrent	Fever episodes of 5 days, 24/ year, with aphthous stomatitis, abdominal pain, diarrhea, fatigue, malaise and occasional episodes of exudative and erythematous pharyngitis, urticarial rash, vomiting and mood disorders. Anal/perianal ulcers with or without fever.		–	Seizures.
	L265R	V377I	4	1	Recurrent (2)	Fever episodes of 3 days, 20 /year, with aphthous stomatitis (2), erythematous pharyngitis, arthralgia, myalgia (1) and occasional abdominal pain, diarrhea, lymphadenopathy, conjunctivitis (2), vomiting, splenomegaly, pneumonia and fatigue (1).		Complete response (1) or failure (1) to steroids on-demand. NSAIDs or colchicine on demand failed (1). Partial response to anakinra as maintenance therapy (1).	
				0.2	Continuous and recurrent (2)	Fever episodes of 4 days, 20/year, with aphthous stomatitis, abdominal pain (2), exudative and erythematous pharyngitis, monoarthritis with tenosynovitis (1) and occasional myalgia, vomiting, diarrhea (2), arthralgia, lymphadenopathy and hepatomegaly (1). Headache and fatigue with or without fever (1). Weight loss (1).		Complete response to steroids on-demand (1).	Flexion contractures (1).
	I268T	V377I	24	0.1	Continuous (1)	Occasional vomiting and diarrhea.	No fever.	–	Amyloidosis.
				1	Recurrent (22)	Fever episodes of 6 days, 14/ year, with lymphadenopathy (18), vomiting, abdominal pain (13), diarrhea, aphthous stomatitis (12), fatigue, malaise (10), arthralgia (8), erythematous or exudative pharyngitis, arthritis, headache (7)	Occasional myalgia (6), maculo-papular rash, arthralgia (5), abdominal pain, mood disorders (4), myalgia, lymphadenopathy, aphthous stomatitis, urticarial rash, ulcers at genitalia, erythema nodosum, aseptic peritonitis, chest pain (2), palpable purpura, papulo-pustular lesions, conjunctivitis, constipation, pericarditis, persistent cough, gastrointestinal bleeding (1) with fever. Presence of fatigue (4), weight loss, headache, splenomegaly (3), vomiting, hepatomegaly, aphthous stomatitis, malaise, mood disorders (2), acne, ulcers at genitalia, erythema nodosum, polyarthritis, abdominal pain, diarrhea, constipation, chest pain, pneumonia, persistent cough, urethritis/cystitis (1) with or without fever.	Complete (2) or partial response (6) or failure (2) to NSAIDs on demand. Partial response (2) or failure (1) to NSAIDs as maintenance therapy. Complete (6) or partial (4) response to steroids on demand. Complete (2) or partial response (3) or failure (1) to anakinra. Partial response (3) or failure (1) to etanercept. Partial response to thalidomide (1) and statins (2) that failed in one patient. Complete response to canakinumab (1). Cimetidine failed (2). No response to tonsillectomy (6).	Amyloidosis (3), cranial nerve palsy, seizures (2), peritoneal adhesions, occlusion/sub-occlusion episodes, cranial neuropathy, mental retardation (1)
				2	Continuous and recurrent (1)	Fever episodes of 8 days, 24/year. Occasional maculo-papular rash, arthralgia, myalgia, headache, conjunctivitis, lymphadenopathy, vomiting, abdominal pain and diarrhea with or without fever.		Steroids, colchicine, methotrexate and anakinra failed as mainenance therapy.	Clubbing.
	S272F	V377I	4	3	Recurrent (1)	Fever episodes of 13 days, 13/year, with vomiting, diarrhea, fatigue, malaise and occasional abdominal pain, aphthous stomatitis, arthralgia, myalgia, oligoarthritis, headache and lymphadenopathy. Persistent splenomegaly.		Partial response to NSAIDs and steroids on-demand. Partial response to cyclosporine, tacrolimus and anakinra as mainenance therapy.	Amyloidosis.
				1	Continuous and recurrent (3)	Fever episodes of 8 days, 10/year, with occasional aphthous stomatitis, arthralgia, myalgia, oligoarthritis (2), abdominal pain, diarrhea, constipation, lymphadenopathy, pericarditis, chest pain and pleurisy (1). Persistent hepatosplenomegaly, fatigue, malaise (2) and occasional acne, hemoptysis and persistent cough (1) with or without fever.		Partial response to steroids as mainenance therapy (1). One partial response and one failure with colchicine. Complete response with anakinra and canakinumab (1), failure with etanercept (2).	
	R277C	R277C	1	6	Recurrent	Fever episodes of 4 days, 20/year, with generalized lymphadenopathy. Persistent abdominal pain with recurrent episodes of vomiting, arthralgia, fatigue and malaise with or without fever.		Complete response to the maintenance therapy with etanercept.	Hyperostosis.
Exon 10	V310 L	V310 L	2	3	Continuous and recurrent	Persistent abdominal pain with diarrhea, fatigue and malaise, gastrointestinal bleeding, arthritis (2), bone pain, uveitis, scleritis and episcleritis (1).	No fever. Recurrent episodes of pyoderma gangrenosum, myalgia or myositis (1).	Partial response to steroids, cimetidine (2) and anakinra (1) as maintenance therapy. NSAIDs and methotrexate (2) failed. Partial response or failure with sulphasalazine or infliximab (1).	Flexion contractures.
	V310 M	V377I	2	2	Recurrent	Fever episodes of 4 days, 11/year, with abdominal pain, diarrhea, cervical and painful lymphadenopathy, arthralgia, malaise, erythematous rash (2), aphtous stomatitis, maculo-papular or urticarial or erysipelas-like rash, pseudo-folliculitis, acne, vomiting, myalgia, fatigue, bone pain (1).	Itchy of the hands (1).	Complete (1) or partial (1) response to steroids on-demand. Partial response (1) or failure (1) to tonsillectomy. Colchicine failed (1). Complete response to anakinra (1) as maintenance therapy.	
	L315 V	V377I	1	0.7	Recurrent	Fever episodes of 3 days, 24/year, with aphthous stomatitis, abdominal pain and arthralgia.		On-demand NSAIDs failed. Complete response to steroids. No response to tonsillectomy.	
	G326R	V377I	1	0.3	Recurrent	Fever episodes of 5 days, 12/year. Occasional episodes of aphthous stomatitis, arthralgia, myalgia, abdominal pain, lymphadenopathy, pneumonia, headache and ulcers at genitalia with or without fever.	Sinus pilonidalis.	Partial response to NSAIDs and complete response to steroids on-demand.	
	S329R	V337I	1	2	Recurrent	Fever episodes of 4 days, 12/year, with aphthous stomatitis, arthralgia, myalgia, abdominal pain, vomiting, diarrhea, generalized and painful lymphadenopathy, fatigue, malaise and occasional erythematous pharyngitis, osteitis, pseudofolliculitis.	Occasional episodes of osteitis.	Partial response to NSAIDs on-demand and to etanercept as maintenance therapy.	
	G335A	V377I	1	1	Recurrent	Fever episodes of 5 days, 18/year, with maculo-papular rash, vomiting, diarrhea, cervical lymphadenopathy, occasional arthralgia, abdominal pain and hepatospenomegaly.		On demand Steroids failed. Complete response to etanercept as maintenance therapy.	
	G336S	V377I	2	1	Recurrent	Fever episodes of 4 days, 12/year, with vomiting, abdominal pain, diarrhea and enlarged cervical lymphadenopathy (2).	Episodes of arthralgia or retinal vasculitis with or without fever (1).	Complete response to steroids on-demand and as maintenance therapy (1).	
	G338D	V377I	1	At birth	Recurrent	Fever episodes of 6 days, 24/year, with abdominal pain, macula-papular rash, occasional aphthous stomatitis, arthralgia, diarrhea, lymphadenopathy. Persistent hepatomegaly and splenomegaly with or without fever.		NSAIDs and steroids on-demand and colchicine, anakinra, etanercept or adalimumab failed. Partial response to canakinumab as maintenance therapy.	
Exon 11	H380R	V377I	1	11	Continuous and recurrent	Episodes of 14 days, 12/year, with or without fever, with erythematous or exudative pharyngitis, arthralgia, myalgia, diarrhea, abdominal pain, constipation, cervical painful lymphadenopathy, aseptic peritonitis, conjunctivitis, oligoarthritis. Persistence of fatigue, malaise, mood disorders, chills and weight loss.	Recurrent chest pain with pleurisy.	Partial response to NSAIDs on-demand. Steroids, methotrexate, etanercept and statins failed as as maintenance therapy.	
	R388*	V377I	2	1	Recurrent	Fever episodes of 3 days, 15/year, with aphthous stomatitis, cervical lymphadenopathy, fatigue, malaise, maculo-papular rash and vomiting (2), abdominal pain, diarrhea, hepatomegaly and exudative or erythematous pharyngitis (1).		Partial response to NSAIDs (2) and complete (1) or partial (1) response to steroids on-demand.	
	V377I	V377I	12	2	Recurrent	Fever episodes of 5 days, 11/year, with lymphadenopathy (11), vomiting, abdominal pain (10), aphthous stomatitis (6 only during fever episodes, 4 with or without fever), diarrhea, malaise (9),myalgia, fatigue (8), headache,arthralgia (7), erythematous (4) or exudative (1) pharyngitis, maculo-papular rash (4), mood disorders, vertigo (3),), arthritis, conjunctivitis (2) and ulcers at genitalia (1). Presence of hepatomegaly or urticarial rash (1) with or without fever.		Complete (2) or partial response (3) to NSAIDs on demand that failed in one patient. Complete (1) or partial response (3) to NSAIDs as maintenance therapy. Complete (2) or partial (2) response to steroids on-demand. Partial response to anakinra (2). Non response to adenotonsillectomy (3).	Seizures (2).

*Designate a translation termination codon according to Human Genome Variation Society nomenclature

**Table 4:** Genotype-phenotype associations in patients with TRAPS

Gene region	Mutation	N° pt	Age at onset (years; average, range)	Disease course	Prevalent clinical manifestations (n. of patients)	Less common manifestations (n. of patients)	Treatment response (n. of patients)	Complications (n. of patients)
Intron 2	c.193-14G > A	1	11	Recurrent	Stress-induced fever for 6 days with erythematous pharyngitis, generalized lymphadenopathy, headache, arthralgia, myalgia and abdominal pain.	Photophobia left eye.	Complete response to steroids during the attacks. Failure with colchicine as maintenance. Tonsillectomy partially effective.	
Exon 2	D12E	3	31 (12–33)	Recurrent	Fever or low fever episodes of 5–15 days, 10/year, with arthralgia, myalgia, conjunctivitis and chest pain with pleurisy. One patient with a mutation in the MEFV gene presented pericarditis.		Partial response to NSAIDs on demand (1). Partial response (1) or deterioration (1) with colchicine. Complete response to steroids, except the patient with a MEFV mutation that responded partially. Complete response to etanercept (1).	
	H22Q	2	0	Continous and recurrent	Fever episodes of 8 days, 10–16/year, with arthralgia, myalgia, periorbital edemaand general malaise. Chronic weight loss.		Partial response to steroids on-demand. Colchicine failed. Complete response to anakinra as maintenance therapy (2).	
	H22R	3	6 (1–8)	Recurrent	Fever episodes of 12 days, 10/year, with urticarial rash, myalgia, and conjunctivitis.		Complete response to steroids on-demand. Complete response to anakinra as maintenance therapy.	
	C29F	1	10	Recurrent	Fever episodes of 7 days, 3/year, induced by stress and fatigue, with urticarial rash, arthralgia and myalgia.		NSAIDs failed.	
	C29Y	1	0	Recurrent	Febrile episodes of 10 days, 4/year, with chills at the onset, erythematous pharyngitis and painful bilateral cervical lymphadenopathy.		NSAIDs failed. Partial response to steroids on-demand. Complete response to canakinumab and anakinra.	
	C30F	1	36	Recurrent	Fever episodes of 8 days, > 24/year, with aphthous stomatitis, myalgia, abdominal pain and mono/oligo/poliarthritis.		Partial response to maintenance with Colchicine.	
	C30R	4	1 (0–1)	Recurrent	Febrile episodes regions of 2–20 days, 1–4/year, sometimes induced by infections, with abdominal pain (4), arthralgia, myalgia, diarrhea and maculo-papular rash(3).		Partial response to steroids (3), colchicine (2) or etanercept (3). Complete response to anakira (1) or steroids (1).	
	C30Y	1	2	Continous and recurrent	Low grade fever with fatigue, malaise and weight loss. Fever episodes of 10–15 days, 6/year, induced by stress and travel, with chills at the onset, with maculo-papular rash, arthralgia, myalgia, oligoarthritis and abdominal pain.		Partial response to steroids. Failure with colchicine as maintenance therapy.	
	C33G	1	1	Recurrent	Episodes of arthralgia, myalgia, periorbital edema and pain, abdominal pain, and fever episodes of 16–20 days, 4/year, with regular frequency.	A patient with two mutations in the MEFV gene: E148Q and M694 V.	Partial response to colchicineand complete response to steroids as maintenance therapy.	
	C33Y	12	7 (0–13)	Recurrent	Fever episodes of 7 days, from 2 to more than 24 episodes/year, with myalgia (9) and abdominal pain (10). In 50–30% were also present urticarial rash (6), chest pain, conjunctivitis and weight loss (4).		Partial response to steroids on-demand (6), failed in one patient. Partial response to thalidomide on-demand (1). Partial responses (2) or failure (2) to NSAIDs. Partial response (4) or complete (1) to etanercept. Complete response to anakinra as maintenance therapy (2).	Renal amyloidosis after 40 years of age (3). Fatal sepsis and renal failure at the age of 60 years (1).
Exon 3	T37I	2	6 (1–9)	Recurrent	Fever episodes of 5 days, 10/year, with maculopapular or urticarial rash, arthralgia, myalgia, abdominal pain, chest pain with pericarditis, pleurisy, pneumonia and persistent cough.		Complete response to steroid (1). Complete response to anakinra (1)with compliance problems. Initial response to Etanercept then failed (2). Partial response with NSAIDs (1) or colchicine (1).	Persistence of elevated sierum amyloid type A (2). Renal amyloidosis at the age of 28 years (1).
	Y38S	1	0	Continous and recurrent	Fever episodes of >20 days, 3/year, with chills at the onset, induced by fatigue, infections, stress and traveling, with maculopapular or migratory rash, arthralgia, myalgia, periorbital edema with conjunctivitis and diarrhea. Chronic low fever with fatigue, malaise and weight loss.	Patient with V198 M mutation in the NALP3 gene.	Partial response to steroids on-demand. Complete response to Anakinra as maintenance therapy.	Renal amyloidosis at the age of 76 years.
	L39F	1	6	Recurrent	Febrile episodes, 12/year, with arthralgia, myalgia, polyarthritis, abdominal pain with aseptic peritonitis.		Colchicine failed.	
	D42del	4	5 (1–6)	Continous and recurrent	Fever episodes of 15 days, 10/year, with abdominal pain (5). In 50–30% of patients fevr episodes with chills at the onset, maculo-papular or migratory or urticarial rash, arthralgia, pleuritis with chest pain, headache and periorbital edema, malaise, fatigue and chronic weight loss (2).		Partial response to NSAIDs on-demand (1). Colchicine failed (3). Complete response to Anakinra as maintenance therapy (2). Partial response to steroids, azathioprine or Etanercept (1).	Renal amyloidosis after the age of 40 years (3). Fatal renal failure (1).
	C43G	1	2	Recurrent	Fever episodes of >20 days, 1/year, with chills at the onset, sometimes induced by exercise, with myalgia, myositis, fasciitis, oligoarthritis, periorbital edema, abdominal pain with aseptic peritonitis, painful bilateral cervical lymphadenopathy, scrotal pain and maculo-papular rash.	Seizures.	Partial response to NSAIDs or steroids on-demand. Methotrexate and colchicine failed. Complete response to Canakinumab.	Laparotomy for peritoneal adhesions at the age of 12 years.
	C43R	3	1 (0–1)	Recurrent	Fever episodes of 10 days, 2/year, with chills at the onset, induced by stress, infections, fatigue, exercise, with arthralgia, myalgia, painful periorbital edema and conjunctivitis, abdominal pain, constipation and chest pain.	Ptosis, perianal ulcers (1). Chronic disease course withlow grade fever, fatigue, malaise, mood disorders and weight loss (1). Cataract and visual changes at the age of 31.	Partial response to NSAIDs or steroids on-demand.Complete response to Anakinra.	Renal amyloidosis (1).
	C43S	1	1	Recurrent	Febrile episodes of 20 days, 4/year, sometimes induced by infections, with urticarial rash, migratory rash, arthralgia, myalgia, abdominal and chest pain.		Partial response to NSAIDs or steroids on-demand. Partial response to cimetidine or etanercept as maintenance therapy, stopped because the patient developed esophagitis. Complete response to Anakinra.	
	C43Y	1	4	Recurrent	Episodes of maculo-papular or migratory rash, arthralgia, myalgia, abdominal pain with constipation, sometimes fever of 8 days, 3/year.with aseptic peritonitis.		NSAIDs failed. Partial response to steroids and Colchicine on-demand. Partial response to etanercept and anakinra as maintenance therapy.	Peritoneal adhesions and intestinal occlusions at the age of 35 years. Renal amyloidosis at the age of 41 years. Renal failure at the age of 52 years.
	T50 K	2	2	Continous and recurrent	Fever episodes of 10 days, 4 or >24/year, with arthralgia and myalgia. Chronic low grade fever, fatigue and malaise.		Partial response to etanercept or colchicine. Complete response to steroids.	
	T50 M	16	6 (0–41)	Recurr ent	Fever episodes of 10 days, 5/year, with myalgia (12), arthralgia (11), abdominal pain (11). 50–30% of patients presented bilateral cervical lymphadenopathy (7) andchest pain (6).	Chronic fatigue and malaise (1).	Partial response to steroids or NSAIDs on-demand (5). NSAIDs failed (2). Complete response to steroids (3). Complete response to anakinra (3) or canakinumab (1) as maintenance therapy. Complete (2) or partial (2) response to etanercept. Tonsillectomy failed (2).	
	C52Y	5	3 (0–11)	Recurrent	Fever episodes of 11 days, 7/year, with painful periorbital edema with conjunctivitis (4), arthralgia, myalgia and abdominal pain (3). Two patients presented chills atthe onset and constipation.		NSAIDs fail (1). Partial (2) or complete (1) response to steroids as maintenance therapy. Complete response to anakinra (2) or canakinumab (1)as maintenance therapy.Colchicine (3) and methotrexate (1) failed as maintenance therapy. Partial response (1) or complete (1) to etanercept withskin reactions at the site of injections.	Renal amyloidosis with organ failure after 33 years (2). In a patient paroxysmal atrial fibrillation at age 45.
	C55Y	4	3 (1–18)	Recurrent	Low fever or fever episodes of 20 days, 4/year, with arthralgia, myalgia, abdominal pain (3). In 50–30% of patients the episodes were induced by stress or menstruation, and were associated with urticaria rash, diarrhea, generalized lymphadenopathy and bilateral cervical pain (2).		Partial response to NSAIDs (2) and complete (2) or partial (1) response to steroids on-demand. Colchicine failed (2). Complete response to anakinra as maintenance therapy (2). Complete response to infliximab (1). Appendicectomy (1).	
	S59P	1	49	Recurrent	Fever episodes of 6 days, 3/year, associated with maculo-papular and migratory rash, arthralgias and myalgias, splenomegaly, pericarditis, chest pain, pleurisy, pneumonia and persistent cough.		Partial response to steroids as maintenance therapy.	Osteoporosis atthe age of 62 years.
	F60 L	2	1 e 6	Recurrent	Fever episodes of >20 days, 3/year, with chills at the onset, urticarial rash, arthralgia and abdominal pain.	A patient presented delayed puberty and bone growth.	Complete response to NSAIDs or steroids on-demand. Complete response to anakinra as maintenance therapy.	
	T61 N	2	0	Recurrent	Fever episodes associated with chills at onset, maculopapular rash, arthralgia, myalgia and abdominal pain.		Complete response to NSAIDs or steroids on-demand.	
	N65I	2	0	Recurrent	Fever episodes associated with myalgia, migratory rash, conjunctivitis, abdominal pain and painful generalized lymphadenopathy.	Swelling and erythema with socks-like distribution.		
	H66L	1	0	Recurrent	Febrile episodes of 3 days, 21/years with bilateral lymphadenopathy.		Steroids failed.	
	L67P	1	0	Recurrent	Febrile episodes of >20 days, 3/year, associated with chills at the onset, induced by stress and vaccinations, associated with pseudofolliculitis, migratory urticarial or maculopapular rash, erythematous pharyngitis, arthralgia, oligoarthritis, abdominal and chest pain, vomiting, headache and conjunctivitis.		Partial response to NSAIDs or steroids on-demand.	
	H69fs	1	1	Recurrent	Fever episodes of 12–24 h, 12/years, with urticarial rash, arthralgia, myalgia, conjunctivitis, periorbital and abdominal pain.		Complete response to NSAIDs on-demand. Steroids failed. Partial response to sulfasalazine as maintenance therapy.	
	C73R	1	0	Recurrent	Fever episodes of 10 days, 14/year, with arthralgia, myalgia, and abdominal pain with constipation.		Partial response to NSAIDs and complete response to steroids on-demand.	
	C73W	2	3 (2–4)	Continous	Myalgia and abdominal pain.		Complete response to NSAIDs on-demand. Complete response to colchicine as maintenance therapy.	
Exon 4	C88Y	1	7	Recurrent	Feverepisodes, 2/year, with chills at the onset and abdominal pain.		Complete response to steroids on-demand.	
	R92P	1	19	Recurrent	Fever episodes of >20 days with arthralgia, myalgia, abdominal pain with constipation, and chest pain with pleurisy.	Abscessesin the groin, armpits, vulva and back after the age of 50 years.	Partial response to steroids and complete response to anakinra as maintenance therapy.Azathioprine failed.	Renal amyloidosis.
	V95 M	2	31 (30–32)	Recurrent	Fever episodes of 11 days, 6/year. A patient presented aphthous stomatitis, maculo-papular rash, arthralgia, myalgia, fasciitis, conjunctivitis and abdominal pain.A patient presented chest pain, pericarditis, pleurisy.		Partial response to NSAIDs and steroids on-demand(2).Complete response to Anakinra as maintenance therapy (1).	
	C96Y	1	30	Recurrent	Episodes of conjunctivitis, fever, oligoarthritis and pleurisy.		Partial response to colchicine as maintenance therapy.	
	Y103_R104del	1	25	Recurrent	Fever episodes of of 7 days, 4/year, with chest pain and pericarditis.		Partial response to NSAIDs, steroids or colchicine.Complete response to etanercept.	
	E109A	2	42 e 1	Recurrent	The patient with the pediatric onset presentedfever episodes of 3 days, 17/year, with erythematous pharyngitis, cervical lymphadenopathy and periorbital edema. The patient with the adult onset presented headache and myalgia associated with fever episodes induced by stress.		The patient with pediatric onset presented partial response to colchicineas maintenance therapy.	
	C114W	1	38	Recurrent	Fever episodes of 8 days, 4/year, induced by menstruation, with erythematous or exudative pharyngitis, vomiting, abdominal pain and constipation.	The patient presented the E148Q and P369S mutations in the MEFV gene.		
	N116S	1	21	Recurrent	Fever episodes of 5 days, 3/year, induced by stress, arthralgia, myalgia, abdominal pain and diarrhea.		Partial response to NSAIDs on-demand.	
Exon 6	L167_G175del	1	6	Recurrent	Fever episodes of 5 days, 2/year, with chills at the onset, induced by fatigue, associated with erythematous pharyngitis, arthralgia, myalgia, painful periorbital edema, conjunctivitis, nausea, abdominal pain andpainful bilateral cervical lymphadenopathy.		Complete response to steroids on-demand. Partial response to anakinra as maintenance therapy.	Renal amyloidosis at the age of 18 years. Renal transplantation at the age of 24 years with an increased number of the fever episodes.

**Table 5:** Genotype-phenotype associations in patients with CAPS

Gene region	Mutation	N° pz	Age of onset (years; average, range)	Disease course (n. patients)	Prevalent clinical manifestations (n. of patients)	Less common manifestations (n. of patients)	Treatment response (n. of patients)	Complications (n. of patients)
Exon 3	R260L	1	8 m	Continuous	Presence of persistent urticarial rash, arthralgia, monoarthritis and morning headache. Occasional fever episodes associated with fatigue.	Sometimes presence of abdominal pain.	Colchicina failed. Complete response to anakinra as maintenancetherapy.	Hearing loss.
	R260W	32	2 (0–24)	Continuous(12)	Presence of persistent urticarial rash (12), associated with arthralgia (7), myalgia (5), conjunctivitis (7), malaise and fatigue (4) and headache (4). Sometimes presence of fever (4).	Occasional episodes of maculo-papular rash, arthritis, (2), aphthous stomatitis, scleritis, papilledema and abdominal pain, conjunctivitis, pleurisy (1).	Partial response to NSAIDson-demand (2) and NSAIDs (1) or steroids (2) as maintenance therapy. Complete (6) or partial (4) response to anakinra or complete (4) or partial (1) response to canakinumeb as maintenance therapy.	Clubbing (1), amyloidosis (1), hearing loss (2), infertility (1).
				Recurrent (16)	Fever episodes of variable length (from 12 h to 5 days), 2 to more than 24/year, characterized by urticarial rash, conjunctivitis (16), arthralgia (14), myalgia (12), headache, fatigue (11), arthritis (8), malaise and livedo reticularis (5).	Occasional papilledema (2), aphthous stomatitis, feet oedema, acanthosis nigricans, bone pain, periorbital oedema, lymphadenopathy, seizures and cystitis/urethritis (1).	Complete (2) or partial (1) response to NSAIDson-demand and partial (2) or failure (1) as maintenance therapy. Partial response to colchicine (2). Cyclophosphamide and cyclosporine failed (1). Complete (2) or partial (1) response to anakinra. Complete response to canakinumab (11).	Clubbing (4) and amyloidosis (2). Hearing loss (5). Infertility (5). Delay in language acquisition (1), facial nerve palsy (1), stammering (1), delayed puberty (1).
				Continuous and recurrent (4)	Presence of persistent urticarial rash, associated with episodes of fever with arthralgia (4), livedo reticularis, myalgia, conjunctivitis and headache (3).	Xerophthalmia (2), papilledema, aseptic meningitis (1), telangectasia (1).	NSAIDs (2),steroids (1) and colchicine (1)failed. Complete (1) or partial (1) response to anakinra. Complete response to canakinumab (3) as maintenance therapy.	Clubbing (3), hearing loss (3), cerebellar syndrome with white matter lesions (1), arterial hypertension (1). Infertility (1).
	D303N	5	1 (0–2)	Continuous (1)	Presence of constant fever associated with fatigue, urticarial rash, monoarthritis, tenosynovitis and sometimes presence of hyperpyrexia, malaise, mood alterations, aphthous stomatitis, arthralgia, oligoarthritis, conjunctivitis and papilledema.	Occasional episodes of anterior uveitis with or without fever.	Partial response to NSAIDs and steroids on-demand. Partial response to methotrexate, etanercept or adalimumab as maintenance therapy, but worsening of the uveitis. Complete response to anakinra.	Severe impaired vision.
				Recurrent (3)	Fever episodes of 8 days. Chronic papilledema (1) and sometimes urticarial (3) and maculo-papular (2) rash, arthralgia (3), oligoarthritis (2), generalized lymphadenopathy (2), headache (2) and aseptic meningitis (2).	Occasional episodes of pseudofolliculitis, bone pain, vaginitis, abdominal pain and diarrhea (1) with or without fever.	Partial response to NSAIDS and steroids on-demand (1). Complete response to anakinra (2) as maintenance therapy. Complete (1) and partial (1) response to canakinumab.	Bone alterations and frontal bossing (1), development of hydrocephalus (1) and hearing loss (2).
				Continuous and recurrent (1)	Recurrent fever episodes of 12–24 h, associated with occasional arthralgia. Moreover, constant presence of urticarial rash and sometimes of fatigue, erythematous pharyngitis, oligoarthritis, conjunctivitis, keratitis, headache, aseptic meningitis and papilledema with or without fever.		Partial response to NSAIDson-demand. Complete response to anakinra as maintenance therapy.	Hearing loss.
	G307 V	2	6 m	Continuous	Persistence of urticarial rash, arthralgia, myalgia, bone pain, arthritis, papilledema, headache, aseptic meningitis.		NSAIDs or steroids failed. Complete response to anakinra or canakinumab.	Patellar overgrowth and hypertrophy of tibia metaphysis.
				Recurrent	Fever episodes of variable length, 3/year. Occasional presence of urticarial rash, arthralgia, oligoarthritis, papilledema and headache, with or without fever.	Episodes of palpable purpura with or without fever.	Partial response to NSAIDs on-demand.	Hydrocephalus and ventricular dilatation.
	E311K	9	3 (0–5)	Continuous (4)	Occasional presence of urticarial rash (4), aphthous stomatitis (3), arthralgia (4), myalgia (1), bone pain (1), monoarthritis (2), oligoarthritis (1), conjunctivitis (4), headache (3) and fatigue (2).	Occasional abdominal pain (1).	Partial response to anakinra (3). Partial (2) or complete (2) response to canakinumab (2).	Hearing loss (2).
				Recurrent (4)	No fever episodes. Presence of persistent arthralgia (4) and occasional urticarial rash (2), headache (2) and vertigo (1). Persistent (2) and occasional (2) conjunctivitis.	Occasional episodes of thoracic pain and pericarditis (1).	Complete response to steroids (1)on-demand.Complete (3) or partial(1) response to anakinra and partial response to canakinumab (1) as maintenance therapy.	Hearing loss (3).
				Continuous and recurrent (1)	Fever episodes of 2 days. Presence of occasional urticarial rash, aphthous stomatitis, arthralgia, oligo-polyarthritis, conjunctivitis, malaise, fatigue and mood alterations with or without fever.	Occasional abdominal pain.	Partial response to anakinra or canakinumab.	Gut perforation, peritoneal adhesion and gut sub/occlusion.
	I334V	1	At birth	Continuous and recurrent	Fever episodes of 7 days, associated with urticarial rash, arthritis, uveitis anterior, papilledema, vomiting, headache, aseptic meningitis.		On-demandNSAIDs failed. Partial response to steroids, anakinra or canakinumabas maintenance therapy, while methotrexate failed.	Impaired vision, mental retardation, hearing loss.
	T348 M	21	At birth (0–2)	Continuous (12)	Presence of urticarial rash (11), conjunctivitis (10) and headache (6). Sometimes presence of fever (7).	Occasional presence of aphthous stomatitis (1), maculo-papular rash (3), arthralgia (3), myalgia (2), bone pain (1), arthritis (4), anterior uveitis (1), papilledema (3), abdominal pain and gastrointestinal bleeding (1), lymphadenopathy (1), splenomegaly (1), hepatomegaly (1), aseptic meningitis (3).	Complete (5) or partial (1) response to anakinraand complete response to canakinumab (10).	Hearing loss (8).
				Recurrent (4)	Episodes with fever (3), urticarial rash (4), aphthous stomatitis (3), maculo-papular rash (2), arthralgia (4), myalgia (1), arthritis (3), conjunctivitis (2), papilledema (3), lymphadenopathy (1), splenomegaly (1), hepatomegaly (1), headache (3), aseptic meningitis (2), fatigue (2), mood alterations (1) and malaise (1).	Presence of occasional livedo reticularis (1).	Partial response to NSAID on-demand and complete response to steroids (1). Complete response to anakinra (3) or canakinumab (2) as maintenance therapy.	Bone alterations and frontal bossing (2), hearing loss (2).
				Continuous and recurrent (5)	Presence of urticarial rash (5), arthralgia (3), myalgia (4) and headache (3). Some patients present fever (3).	Occasional presence of maculo-papular rash, livedo reticularis, conjunctivitis, episcleritis, papilledema, abdominale pain and vomiting, aseptic meningitis, hypotonia and vertigo (1).	Colchicine (1) failed. Complete response to anakinra (2). Complete (4) or partial (1) response to canakinumab. Adenotonsillectomy failed (1).	Presence of bone alterations (2), frontal bossing (2), clubbing (1), cataract (1), glaucoma (1) and hearing loss (2).
	P350L	2	1	Continuous	Persistent urticarial rash, arthralgia, fatigue and weight loss (2).	Aphthous stomatitis, erythematous pharyngitis, acne, erythema nodosum, oligoarthritis, conjunctivitis, headache,uveitis, scleritis, papilledema, abdominal and chest pain with diarrhea (1).	NSAIDs or methotrexate failxed (1). Partial response to anakinra (1) as maintenance therapy.	Hearing loss (2), impaired vision (1), delayed puberty and bone age (1).
	V351 M	1	At birth	Continuous and recurrent	Fever episodes of 12–24 h. Persistent urticarial rash, with or without fever, and occasional arthralgia, conjunctivitis, papilledema, vomiting, headache and aseptic meningitis.		Partial response to anakinra as maintenance therapy.	Presence of bone alterations and frontal bossing, hearing loss, hydrocephalus and severe mental delay.
	A352V	1	5 m	Continuous e recurrent	Fever episodes of 2 days, 2/year, with fatigue and malaise. Occasional presence of urticarial rash, conjunctivitis and fatigue, with or without fever.		Complete response to canakinumab as maintenance therapy.	
	E354D	1	2 m	Continuous	Persistent presence of urticarial rash, arthralgia, lymphadenopathy and malaise, not associated with recurrent fever episodes.		Complete response to anakinra as maintenance therapy.	
	H358R	1	At birth	Continuous and recurrent	Fever episodes of 16–20 days, 8/year. Constant presence of maculo-papular rash, sometimes associated with aphthous stomatitis and aseptic meningitis, with or without fever.	Vomiting and diarrhea with orwithout fever.	Complete response to anakinra or canakinumab as maintenance therapy. Dapsone failed.	Hearing loss and severe mental retardation.
	K375E	1	9 m	Recurrent	Fever episode of 2 days, >24/year, with urticarial rash, aphthous stomatitis, arthro-myalgia, periorbital oedema, conjunctivitis, headache and fatigue.		Partial response to steroids or anakinra on-demand. Complete response to canakinumab as maintenance therapy.	
	M406I	1	5 m	Recurrent	Fever episode of 3 days, >24/year. Occasional presence of urticarial and maculo-papular rash, erythematous pharyngitis, aphthous stomatitis, arthro-myalgia, papilledema, generalized lymphadenopathy, fatigue, malaise, mood alterations and headache.		Complete response to canakinumab as maintenance therapy.	Bone alterations and frontal bossing. Moderate mental retardation. Delayed puberty.
	T436A	1	At birth	Continuous	Persistent presence of urticarial rash, arthralgia, headache and optic neuritis. Sometimes conjunctivitis.		Partial response to steroids. Complete response to anakinra.	Hearing loss, infertility and bone age delayed.
	T436I	1	At birth	Continuous and recurrent	Fever episodes of 12–24 h, >24/year, with fatigue, mood alterations, malaise and occasional urticarial rash, conjunctivitis, periorbital pain and morning headache. Occasional aphthous stomatitis, exudative and erythematous pharyngitis, maculo-papular rash, arthralgia, myalgia, papilledema, headache and persistent polyarthritis, with or without fever.	Severe cutaneous involvement with pathergia.	Partial response to NSAIDson-demand and to canakinumab as maintenance therapy.	Flexion contractures, cataract, optic neuritis and optic nerve atrophy, band keratopathy and impaired vision. Hearing loss.
	A439V	14	5 (0–20)	Continuous (5)	Persistent presence of urticarial rash (4), arthralgia (3), myalgia (1), oligoarthritis (1). Sometimes presence of aphthous stomatitis (3), headache (2) and fever (1). Persistent (1) and episodic (2) conjunctivitis.	Presence of xerophthalmia and xerostomia, diarrhea (1).	Complete response to anakinra (4) or canakinumab (1) as maintenance therapy.	Tinnitus (1), renal amyloidosis (1).
				Recurrent (8)	Two patients present persistent fever. Presence of urticarial rash (8), in some patients only with fever, arthralgia (7), myalgia (3), arthritis (3), conjunctivitis (6), fatigue and malaise (3) and headache (5).	Presence of anterior uveitis and periorbital pain, episodes of uretritis and frequent episodes of urinarial tract infections (1).	Partial (1) or complete (2) response to anakinra as maintenance therapy. Partial (1) or complete (6) response to canakinumab.	Clubbing (2), bone alterations and frontal bossing (1). Diverticulitis (1).
				Continuous and recurrent (1)	Fever episodes of 5 days, 12/year. Occasional presence of urticarial and maculo-papular rash, arthralgia, myalgia, arthritis, conjunctivitis, malaise and fatigue with or without fever.	Abdominal pain without fever.	Partial response to steroids on-demand. Partial response to anakinra as maintenance therapy.	Cataract, glaucoma, hearing loss and peripheric polyneuropathy.
	N477 K	1	2	Recurrent	Fever episodes ofless then 12 h, >24/year. Occasional urticarial rash, arthralgia, papilledema, generalized lymphadenopathy, hepatomegaly, headache, failure, malaise and mood alterations with or without fever. Constant presence of splenomegaly.	Occasional thoracic pain without fever.	Complete response to anakinra as maintenance therapy.	Clubbing and frontal bossing, impaired vision, severe mental retardation and hearing loss. Cardiomyopathy. Delayed puberty.
	Y572F	1	At birth	Recurrent	Fever episodes, >24/year, with urticarial rash, mood disorders and weight loss.		Partial response to NSAIDson-demand. Complete response to anakinra as maintenance therapy.	Flexion contractures, hydrocephalus.
	F523C	1	1	Continuous and recurrent	Fever episodes of 12–24 h, >24/year,with conjunctivitis, headache, fatigue and malaise. Occasional urticarial rash, arthralgia and myalgia.		Partial response to NSAIDson-demand. Complete response to canakinumab as maintenance therapy.	Frontal bossing and clubbing.
	G569R	1	1 m	Recurrent	Fever episodes of less then <12 h, >24/year, with malaise and fatigue. Occasional migratory urticarial and maculo-papular rash, arthralgia, conjunctivitis, lymphadenopathy and aseptic meningitis.	Occasional anterior and posterior uveitis.	On-demand NSAIDs failed. Complete response to anakinra as maintenance therapy.	Hydrocephalus and hearing loss.
	Y570F	1	1 m	Continuous	Persistent migratory maculo-papular rash, arthralgia, fatigue, malaise, oligoarthritis, conjunctivitis, papilledema, hepatomegaly and splenomegaly.	Occasional episodes of vomiting.	Partial response to NSAIDson-demand. Partial response to steroids, methotrexate, biphosphonates, cimetidine, rhGH, acetazolamide, anakinra and canakinumab as maintenance therapy.	Optic nerve atrophy and impaired vision. Hearing loss and delayed neuromotor development.
	I572F	1	At birth	Continuous and recurrent	Fever episodes of > 20 days. Occasional urticarial rash, arthralgia, oligoarthritis, papilledema, hepatomegaly, splenomegaly and headache.	Occasional seizures with or without fever.	Complete response to anakinra or canakinumab as maintenance therapy.	Bone alterations with frontal bossing, optic nerve atrophy with severe vision impairment, hydrocephalus and hearing loss. Severe mental retardation. Delayed puberty and bone age.
	E627G	1	3 m	Recurrent	Occasional urticarial rash, arthralgia, myalgia, abdominal pain, constipation with and without fever.		Complete response to anakinra as maintenance therapy.	
	M662 T	2	6 m	Continuous and recurrent	Fever episodes of 5 days, >24 per year, with urticarial rash, aseptic meningitis and generalized lymphadenopathy (2). Persistent papilledema (2) and headache (1).	Uveitis anterior, chest pain, pericarditis, erythema nodosum.	Partial response to NSAIDs or steroids on-demand (1). Partial response to colchicine (1), anakinra (2) or canakinumab (1) as maintenance therapy. Methotrexate or etanercept (1) failed.	Bone alterations and frontal bossing, hearing loss (2), moderate optic nerve atrophy, hydrocephalus (1).
	S710C	1	2	Recurrent	Fever episodes of 7 days, 2/year, sometimes associated with urticarial and maculo-papular rash, fatigue, malaise, conjunctivitis and cervical bilateral lymphadenopathy. Occasional arthralgia, myalgia, monoarthritis and splenomegaly.		Partial response to NSAIDs and steroids on-demand. Partial response to methotrexate and complete response to anakinra or canakinumab as maintenance therapy.	
Exon 9	L1016F	1	3 m	Continuous	Persistence of fever, urticarial rash, fatigue and weight loss.			Hearing loss.

**Table 6:** Patients with an incomplete or not confirmatory genotype

Disease	Gene region	First variant	Second variant	N° pt	Age at onset (years; average, range)	Disease course (n. of patients)	Prevalent clinical manifestations (n. of patients)	Less common manifestations (n. of patients)	Treatment response (n. of patients)	Complications (n. of patients)
FMF	Exon 2	S141I	–	1	7	Recurrent	Fever episodes of 1 day, 2/year, with abdominal pain.		Complete response to steroids or NSAIDs.	
		E148V	–	1	7	Recurrent	Fever episodes of 3 days, with myalgia, arthralgia, mono/oligo/poliarthritis with tenosynovitis, abdominal pain.		Partial response to NSAID on demand. Complete response to cochicine.	
			E148Q	1	0	Continuos and recurrent	Fever episodes of 3 days with aphtous stomatitis, ulcers at genitalia, arthralgia associated with monoarthitis and abdominal pain with vomiting, diarrhea or costipation. Chronic fever and weight loss.		Ppartial response to NSAIDs on demand. Partial response to colchicine, cimetidine or steroids as maintenance therapy. Cotrimoxazole and infusions of immunoglobulins failed.	
		E148Q	–	7	5 (0–11)	Recurrent	Fever episodes of 4 days, 12/year, with abdominal pain (6), arthralgia (5), myalgia, weight loss (4) and vomiting (3).	Enlarged cervical lymphonodes, exudative pharyngitis, urticarial rash, oligoarthritis, orchitis/gonadal pain (2), aphtous stomatitis, palpable purpura, diarrhea, chest pain, persistent cough, headache, seizures, vulvovaginitis, monoarthritis, conjunctivitis (1)	Partial (1) or complete (1) response to NSAIDs or steroids. Complete (1) or partial (1) response to colchicine as maintenance therapy that failed in two patients. Complete response to canakinumab (1). Methotrexate failed (1).	
			E148Q	6	2 (1–10)	Recurrent	Fever (3) or low fever (6) episodes of 2–3 days, 16/year, with abdominal pain (5), myalgia, arthralgia (4), vomiting, diarrhea (3), generalized lymphonodes enlargement and chest pain (2).	Aphtous stomatitis, monoarthitis, acne, pseudofolliculitis, enlarged cervical lymphonodes, flexon contractures, osteolytic lesions and sacroileitis, hepatomegaly and splenomegaly, pericarditis, headache, urethritis/cititis, gonal pain, epidydimitis and weight loss (1).	Partial response to NSAIDs (2). Steroids failed as maintenance therapy (1). Complete response to azathioprine or colchicina or sulphalazine and methotrexate (1).	
			R202Q^a^	1	40	Recurrent	Low fever episodes of 2 days, 10/year, with aphtous stomatitis, arthralgia, myalgia, generalized lymphonodes enlargement, hepatosplenomegaly and chest pain.		Partial response to NSAIDs on demand. Complete response to colchicine as maintenance therapy.	
			P369S	3	3 (2–10)	Recurrent	Fever or low fever episodes of 4 days, 12/year, with regular pattern and chills at fever onset, abdominal pain, myalgia, arthralgia, vomiting and diarrhea (3).	Poliarthritis or bone pain (1).	Partial response to NSAIDs on demand (2). Partial response to steroids as maintenance therapy (2). Complete response to colchicine (3).	
		P180R	R202Q^a^	1	3	Continuos and recurrent	Fever or low fever episodes of 2 days, 20/year, with regular pattern, characterized by maculo-papular or urticarial rash, arthralgia, mono/oligo/polyarthritis and headache.		Partial response to steroids or cochicine as maintenance therapy. Methotrexate failed.	
		R202Q^	–	4	4 (1–8)	Recurrent	Fever episodes of 4 days, 12/year, with abdominal pain (4) with vomiting or diarrhea, fatigue, chills at fever onset with mood disorders (3), aphtous stomatitis, exudative pharyngitis, malaise, arthalgia, enlarged cervical lymphonodes (2).	Painful enlarged generalized lymphonodes, myalgia, bone pain and bone age delay (1).	Complete or partial response to NSAIDs or steorids. Partial response to colchicine as maintenance therapy (1), that failed in one patient. Partial response to anakinra (1).	
			M694 V	7	9 (4–30)	Recurrent	Fever episodes of 2 days, 8/year, with abdominal pain (6), arthralgia and myalgia (4).	Bilateral cervical enlargement lymphonodes (2), generalized painful lymphonodes, chills at fever onset, urticarial rash, diarrhea (1).	Partial (3) or complete (1) response to NSAIDs on demand. Partial (1) or complete (1) response to steroids. Complete response to colchicine as maintenance therapy.	
	Exon 3	P369S	–	1	At birth	Recurrent	Fever episodes without a specific pattern of recurrency, with abdominal pain, diarrhea, aphtous stomatitis, erythematous pharyngitis, arthralgia, myalgia, monoarthritis, conjunctivitis		Complete response to NSAIDs on demand.	
			R408Q	2	46 (25–67)	Recurrent	Fever episodes of 2–3 days, 8/year, with chills, athralgia, myalgia (2), chest pain, pericarditis, pleurisy, erythematous pharyngitis, erysipelas-like erythema, conjuntivitis, vomiting, abdominal pain and diarrhea (1).		Partial response to NSAIDs on demand. Steroids failed. Complete or partial response to colchicine.	
			A744S	1	8	Recurrent	Fever episodes of 4 days, 3/year, stress and travel induced, with exudative pharyngitis and arthralgia.		Partial response to NSAIDs on demand. Partial response to colchicine as maintenance therapy.	
	Exon 5	D510D	–	2	4 (3–5)	Recurrent	Fever episodes of 2 days, 4–24/year, induced by cold and fatigue (1), with chills at fever onset, athralgia and abdominal pain (2), headache, constipation, arthralgia, myalgia, exudative pharyngitis, maculo-papular rash, fatigue (1).		Complete response to steroids on demand (1) or canakinumab as maintenance therapy (1).	Neurosensorial hearing loss (1).
	Exon 9	I591T	–	1	13	Recurrent	Fever episodes of 4 days, 15/year, with abdominal and chest pain, urticarial rash, oligoarthritis, headache, arthralgia, myalgia, fatigue and malaise.		Partial response to colchicine as maintenance therapy.	
	Exon 10	I641F	–	2	3 (0–5)	Recurrent	Fever episodes of 2–3 days characterized by constipation, enlarged cervical lymphonodes, abdominal pain (2), chest pain, pleurisy, hepatosplenomegaly, vomiting, chills, monoarthritis, arthralgia and headache (1). Triggers: muscolar exercise (1).		Partial response to NSAIDs on demand (1). Complete response to colchicine (2).	
		M680IGC	–	5	3 (0–8)	Recurrent	Fever episodes of 3 days, 14/year with abdominal pain (4), arthralgia, myalgia, (3) and vomiting (2).	Exudative pharyngitis, chills, constipation, diarrhea, chest and bone pain and myositis (1). Fever episodes were induced by stress or menstrations (1).	Complete (1) or partial (2) response to NSAIDs on demand that failed in one patient. Non response to tonsillectomy (1). Complete (3) or partial (1) response to colchicine.	
		M680 L	–	2	2	Recurrent	Fever episodes of 1–2 days, >24/year, with regular pattern, associated with abdominal pain (2) and diarrhea (1).		Complete response to colchicine as maintenance therapy (1) or on demand (1).	
		M694I	–	1	0	Recurrent	Fever episodes of 2 days, >24/year, with chills at onset, associated with aphtous stomatitis, exudative pharyngitis with bilateral cervical or generalized enlarged lymphonodes, maculopapular rash, arthralgia, monoarthritis, chest pain, headache, costipation and abdominal pain.		Complete response to colchicine as maintenance therapy.	
		M694 V	–	48	3 (0–36)	Recurrent	Fever episodes of 3 days, 11/year, with abdominal pain (42), arthralgia (27), myalgia (23), chest pain (16) and diarrhea (15).	Vomiting (12), costipation (10), monoarthritis (7), erysipelas like erythema, headache (6), enlargement generalized and/or cervical painful lymphonodes, exudative pharyngitis (4), gastrointestinal ulcers, aseptic peritonitis, persistent cough, aphtous stomatitis, headache in the morning (3), palpable porpora, bone pain, pericarditis, pleuritis, maculopapular rash (2), gastrointestinal bleeding, oligoarthritis, poyarthritis, migratory or urticarial rash, psoriasis, pneumonia, conjunctivitis, oligoarthritis, periorbital pain and seizures (1).	Partial (11) or complete (6) response to NSAIDs. Partial (4) or complete (7) response to steroids. Partial (17) or complete (22) responseo to colchicine. Complete (2) or partial (2) response to anakinra. Complete response to canakinumab (1). Tonsillectomy failed (1)	Occlusion, peripheral neuropathy, cerebellar syndrome, megalocephaly (1).
		K695R	–	4	7 (3–12)	Recurrent	Fever episodes of 9 days, 12/year, with abdominal pain, mono/oligoarthritis, myalgia, generalized enlargement of lymphonodes (3), arthralgia, vomiting, headache, chest pain, fatigue and malaise(2).	Exudative pharyngitis, erysipelas-like and perioral erythema, herpetiform dermatitis, bone pain, hepatosplenomegaly, aseptic peritonitis diarrhea and gonadal pain (1). Fever episodes were induced by stress or fatigue (1).	Partial response to NSAIDs (1) or steroids (2) on demand. Steroids failed (1). Complete response to methotrexate (1). Complete response to colchicine (2) or anakinra (1) as maintenance therapy.	
		V726A	–	2	21 (12–30)	Recurrent	Fever episodes of 1–2 days, 14/year, with arthralgia and abdominal pain (2).	Aphtous stomatitis, exudative pharyngitis, urticarial rash, erythematous pharyngitis, myalgia, bone pain, diarrhea, painful enlarged cervical lymphonodes, urethritis/cistitis, headache and vertigo (1).	Complete response to colchicine (2).	
		A744S	–	2	2 (1–3)	Recurrent	Fever episodes of 3 days, 4–5/year, with abdominal pain, wight loss (2),arthralgia, myalgia, diarrhea, vomiting, exudative pharyngitis, aphtous stomatitis, enlarged cervical lymphadenopathy, seizures, fatigue and malaise (1).		Partial response to NSAIDs or steroids on demand (1). Complete (1) or partial (1) response to colchicine as maintenance therapy.	
		R761H	–	3	7 (1–13)	Recurrent	Fever or low fever episodes of 3 days, 11/year, with arthralgia, myalgia (3), generalized painful enlargement of lymphonodes, headache (2), constipation, mono/oligoarthritis, abdominal pain, chest pain, seizure, fatigue, malaise and weight loss(1).		Partial response to NSAIDs (2) or steroids (2) on demand. Complete response to colchicine (1) or steroids (1). Partial response to methotrexate (1). NO response to tonsillectomy (1).	–
MKD	Exon 2	H20Q	–	1	2 m	Continous and recurrent	Fever episodes of 4 days, 12/year, with lymphadenopathy, fatigue, malaise and mood disorders. Occasional vomiting, abdominal pain, diarrhea, gastrointestinal bleeding, perianal ulcers, maculo-papular and migratory rash, myalgia with or without fever.	Persistent constipation, polyarthritis and occasional myositis and tenosynovitis with or without fever.	Partial response to steroids, biphosphonates and anakinra on-demand. On-demand NSAIDs failed. Partial response to etanercept as maintenance therapy. Methotrexate and cyclosporine failed.	Bone alterations and deformity, flexion contractures and osteoporosis.
	Exon 5	P165L	–	1	6	Recurrent	Fever episodes of 5 days. Occasional palpable purpura, arthralgia, myalgia, vomiting, abdominal pain, diarrhea and lymphadenopathy with or without fever.	Occasional urethritis/cystitis with or without fever.	Partial response to NSAIDs on-demand.	
	Exon 7	R215*	–	1	2	Recurrent	Fever episodes of 20 days, 5/year, with lymphadenopathy and persistent arthralgia, myalgia and polyarthritis.		Partial response to NSAIDs as maintenance therapy.	
	Exon 11	V377I	–	11	3	Recurrent	Fever episodes of 5 days, 9/year, with abdominal pain, diarrhea, lymphadenopathy (10), vomiting (9), fatigue, myalgia, malaise (7), arthralgia (6), arthritis (4), exudative (1) or erythematous pharyngitis, maculo-papular rash, headache, aphthous stomatitis, and mood disorder (1). Occasional episodes of palpable purpura (2), erythema nodosum, aseptic peritonitis, pericarditis (1) during the fever episodes. Splenomegaly (2), hepatomegaly, panniculitis, persistent cough and perianal ulcers (1) with or without fever.	Episodes of erythematous and itchy rash during fever episodes (1) or without fever (2). Recurrent pericarditis (1).	Partial response to NSAID (8). Partial (1) or complete (4) response to stereoids and partial (4) or complete (1) response to anakinra on-demand. Failure or partial response to statins (1). colchicine failed (2). Partial response to etanercept (1) or anakinra (1).	Peritoneal adhesions (1).
TRAPS	Intron 2	c.194-15C > T	–	1	4	Recurrent	Fever or low fever episodes of 9 days, 2/year, with aphthous stomatitis, myalgia, abdominal pain, fatigue and cervical unilateral lymphadenopathy.		Partial response to steroids as maintenance therapy.	Renal failure at the age of 15 years.
	Intron 4	c.472 + 6C > T	–	2	43 e 6	Recurrent	Fever episodes of 10–20 days or more, 6–10/year, with arthralgia, myalgia, abdominal and chest pain.		NSAIDs and colchicine failed (1). Partial response to etanercept (2).	
	Intron 6	c.626-32G > T	–	1	2	Recurrent	Fever episodes of 5 days, 10/year, with chills at the onset, and maculo-papular rash, arthralgia, myalgia, abdominal pain, aseptic peritonitis, vomiting, diarrhea, chest pain, testicular pain and pericarditis.		Partial response to NSAIDs and steroids. Colchicine failed.	Appendectomy at the age of 32 years.
	Exon 3	P46L	–	5	6 (2–63)	Recurrent	Fever episodes of >7 days, 7/year, with urticarial rash and myalgia. 50–30% of patients had erythematous pharyngitis, maculopapular rash, arthralgia, abdominal pain, headache.	Swelling of the scalp, cranial nerve palsy, mental retardation, urethritis/cystitis (1). Persistent fever, fatigue, malaise and weight loss (1).	Complete (1) or partial (1) response to steroids on demand. Etanercept failed (1). Partial response to NSAIDs (1).	Renal amyloidosis at the age of 20 years (1).
	Exon 4	R92Q	–	53	8 (0–53)	Recurrent	Fever episodes of 10 days, 7–8/year, without seasonal changes or regular frequency, with arthralgia (37), myalgia (35), abdominal pain (34). 50–30% patients presented chills and headache at the onset (20), three patients only during the morning.	Papable purpura, chronic rhinitis, visual changes, occlusion, arterial/venous thrombosis and left arm paresthesia, cardiomyopathy (1). Five patients (10%) had chronic course with fever, fatigue, malaise and weight loss.	Complete (9) or patial (11) response to steroids on demand, that failed in one patient. Partial response to NSAIDs (10), that failed in 2 patients. Complete response to infliximab (1) or partial response to colchicine (1) on demand. Complete response to anakinra (3) or etanercept (6) or steroids (3) as maintenance therapy. Partial response to etanercept (6), NSAIDs (5), steroids (2), anakinra or azathioprine or aspirin with tigecycline (1). Partial (2) or complete (1) response to tonsillectomy.	Osteoporosis (3). Sepsis due to Streptococcus pneumoniae during the treatment with etanercept (1).
CAPS	Exon 3	V198 M	–	18	10 (0–45)	Continuous (5)	Presence of arthralgia (5), urticarial rash and myalgia (4).	Occasional episodes of fever (3), maculo-papular rash, palmo-plantar erythema, vomiting, lymphadenopathy and pericarditis (1). Presence of polyarthritis, tenosynovitis, fatigue and conjunctivitis (1). Persistent fever (1).	Partial response to NSAIDs on-demand (1). Partial response to steroids as maintenance therapy (1). Partial response to methotrexate, cyclosporine, infliximab, etanercept, adalimumab, sirolimus and mycophenolate mofetil (1). Complete response to tocilizumab and bisphosphonates as maintenance therapy (1). Partial (1) or complete (3) response to anakinra, which failed in one patient.	Hearing loss (2). Osteoporosis, osteolytic lesions, bone erosions, hyperostosis and bone deformity, cataract, amyloidosis, arterial hypertension, delayed puberty (1).
						Recurrent (10)	Episodes, 12/year, induced by cold (3), sometimes with fever, characterized by arthralgia (9), urticarial or maculo-papular rash (8), oligo/monoarthritis, conjunctivitis (7), headache, malaise, fatigue (6), weight loss, myalgia (5), lachrymal gland involvement, periorbital pain and lymphadenopathy (3).	Occasional bone pain, abdominal pain or polyarthritis (1).	Failure (1) or partial (3) response to NSAIDs on demand. Partial response to steroids (3) on-demand. Methotrexate (2), etanercept or cyclosporine (1) failed as maintenance therapy. Failure (1) or partial (1) response to colchicine. Complete (2) or partial (1) response to anakinra. Partial response to canakinumab (2).	Hearing loss, impaired vision (1).
						Continuous and recurrent (3)	Episode characterized by fever, urticarial rash (3), arthralgia, myalgia, conjunctivitis (2), periorbital pain, papilledema, headache, fatigue and malaise (1).		Partial response to NSAIDs or steroids on-demand (2). Complete response to anakinra as maintenance therapy (1).	Bone alterations, frontal bossing, hearing loss (1).
		Q703K	–	9	16 (0–57)	Continuous (3)	Presence of urticarial rash, arthralgia, fever, conjunctivitis (2) and malaise or mood alterations (1).	Maculo-papular or eczmatous rash, myalgia, aphtous stomatitis and headache (1).	Partial response to NSAIDs on demand (1). Complete response to anakinra or canakinumab as maintenance therapy.	Cranial neuropathy (1).
						Recurrent (6)	Fever episodes of 4 days, 9/year, with arthro-myalgia, maculo-papular or urticarial rash (4), weight loss (3), palpable purpura, erythematous pharyngitis, sometimes exudative, aphthous stomatitis, headache, periorbital oedema and conjunctivitis (2). Recurrent episodes without fever (2).	Abdominal pain, diarrhea, urethritis/cystitis, chest pain (1).	Partial response to NSAIDs on demand (5). Colchicine and adenotonsillectomy failed (1). Complete response to steroids (1).	

^a^ *p*.R202Q of the *MEFV* gene is considered a common and neutral polymorphism that should not be even reported

Among 346 FMF patients, 238 were collected in the Table 2, 112 of which were homozygous and 126 carried more than one heterozygous variant. Unfortunately, in these cases, as we did not know the phasing of the alleles, no confirmatory genotype could be assessed. The most frequent mutations are p.M694 V (192 patients), p.V726A (48), p.M680IGC (43), and p.E148Q (31). One hundred eight patients were classified as FMF despite an incomplete (heterozygous) or not confirmatory (p.R202Q or variants of unknown significance) genotype. The clinical features of these patients are reported in Table 6.

Forty-six variants were reported in 158 TRAPS patients. Of these, 4 variants are in intronic regions, 18 involved cysteine domains and 3 are deletions. The most frequent mutations are p.T50 M (16 patients) and p.C33Y (12). The clinical characteristics of 62 patients carrying variants of unknown significance, namely p.R92Q (53) and p.P46L (5), or intronic variants (4), exept c.193-14G > A, are reported in Table 6.

Among the 133 CAPS patients analyzed, 27 different variants were reported in Table 3. With the exception of the variant p.L1016F, that is located in exon 9, all the other variants are on exon 3. The most frequent variants are p.R260W (32 patients) and p.T348 M (21). The patients carrying variants of unknown significance, namely p.V198 M and p.Q703K, are reported in Table 6.

Finally, 114 MKD patients were reported. Seventeen patients were homozygous and 83 heterozygous for more than one variant, showing 47 different combinations of mutations/deletions (Table 2). The most frequent variants were p.V377I (98 patients) and p.I268T (28). Three deletions are described. The clinical features of 14 patients harbouring one MVK mutation (heterozygous) in combination with an abnormal metabolic test are reported in Table 6.

## Discussion

In the present paper we report the largest collection of data related to genotype-phenotype associations of inherited recurrent fevers. The aim of the present work was to provide clinicians and geneticists, working in the field of autoinflammatory diseases, with a practical tool for the interpretation of the results coming from the genetic analysis and to verify the phenotypes already described with a given genotype.

So far, the Infevers database has been the most commonly used tool for the orientation on the clinical relevance of a given variant detected in HRF genes. Infevers is a registry of all the variants identified in association with Autoinfammatory diseases. At present 1523 variants associated with 30 autoinflammatory diseases are reported. Among them, 317 variants for *MEFV*, 204 for *MVK*, 150 for *TNFRSF1A* and 182 for *NLRP3*. For each variant a number of input datais provided (i.e. location, sequence and protein name of Human Genome Variation Society). In addition, a rough description of the clinical phenotype described in the first patient(s) in which a given variant has been identified is also provided. Due to this limitation, Infevers recommends not to use the database as a tool for genotype-phenotype associations.

A number of other web-based instruments provide information concerning the in-silico prediction of the functional impact of each known variant and its frequency in different populations (e.i. https://varsome.com/, http://www.ensembl.org/index.html, ecc.). In some cases, references to published data concerning the clinical phenotype can be also retrieved. However, these instruments are mainly targeted to geneticists and are not of immediate use for the clinicians in the everyday clinical practice. With the present work, we would like to provide an easy instrument for the evaluation of the genotype-phenotype associations, possibly related to different variants associated with HRF.

One important caveat is that this data set only includes patients with a verified diagnosis of an inherited fever syndrome. Some of these patients carry known low penetrance variants such as p.E148Q and p.P369S in *MEFV*; p.R92Q and p.P46L in *TNFRSF1A*; p.V198 M and p.Q703K in *NLRP3*. These genetic variants are frequent in the healthy population although they are usually over represented in patients referred for investigation of systemic inflammatory symptoms. As these patients enrolled into Eurofever represent only a minority of carriers of these genetics variants, the data presented should not be extrapolated to a decription of the phenotype in unselected patients who have undergone genetic testing. Moreover, it should be emphasized that only a limited proportion of the Eurofever patients carrying these low penetrance variants were selected in the process of validation, mainly because their clinical manifestations and response to treatment were consistent with the related disease.

The tables presented here will be available online, with links to the Eurofever (www.printo.it.eurofever) and Infevers websites. Moreover, a continuous updating of the tables will be performed for any new variant associated with patients enrolled in the Eurofever registry.

## Conclusions

We provide a potentially useful tool for physicians dealing with HRF, namely a registry of genotype-phenotype associations for patients enrolled in the Eurofever registry. This tool is complementary to the Infevers database and will be available at the Eurofever and Infevers websites.

## Abbreviations

CAPS: Cryopyrin-Associated Periodic Syndrome
FMF: Familiar Mediterranean Fever
HRF: Hereditary recurrent fevers
MKD: Mevalonate-Kinase Deficiency
TRAPS: TNF-receptor associated periodic fever syndrome
